# *MTS1* regulates rice plant architecture by mediating phosphoinositide metabolism

**DOI:** 10.1111/pbi.70247

**Published:** 2025-07-08

**Authors:** Di Yu, Shuangshuang Zhao, Liping Sun, Daokuan Guo, Wanxia Jiang, Xin Ma, Ruichao Li, Jun Zou, Lubin Tan

**Affiliations:** ^1^ Frontiers Science Center for Molecular Design Breeding (MOE), Department of Plant Genetics and Breeding, China Agricultural University Beijing China; ^2^ Leibniz Institute of Plant Genetics and Crop Plant Research Seeland Germany; ^3^ State Key Laboratory of Plant Environmental Resilience, College of Biological Sciences, China Agricultural University Beijing China

**Keywords:** phosphoinositide metabolism, gibberellin, plant architecture, rice

## Abstract

Plant architecture is an agronomically important trait affecting grain yield in rice (*Oryza sativa* L.). Here, we identified a gene, *Moderate‐tillering and Semi‐dwarf 1* (*MTS1*), which encodes a type II Inositol polyphosphate 5‐phosphatase (5PTase). Compared to the wild type, the *mts1* mutant exhibited a significant reduction in plant height and an increase in effective tiller number. Further analysis showed that the mutation in *MTS1* impairs the hydrolysis of phosphatidylinositol 4,5‐bisphosphate [PI(4,5)P2], resulting in its aberrant accumulation and subsequent suppression of actin polymerization. Notably, the mutation in *MTS1* specifically affects the response to gibberellin at the reproductive growth stage. Both lipid–protein overlay assay and microscale thermophoresis assay demonstrated that phosphatidylinositol 4‐phosphate (PI4P) interacts with NGR5. PI4P promotes NGR5 degradation, thereby modulating plant architecture. Investigation of yield‐related traits showed that the *mts1* mutant has a similar grain yield per plant to the wild type due to the compensation effect of increasing effective tiller number. These findings suggest that phosphoinositide metabolism plays a critical role in modulating plant architecture in rice and the favourable allele of *MTS1* has potential application value in rice dwarf breeding.

## Introduction

Rice (*Oryza sativa* L.) is a staple food for more than half of the world's population. Plant architecture plays an important role in determining grain yield in rice, through affecting plant density, lodging resistance and population‐level photosynthetic efficiency (Tan *et al*., 2008; Wang *et al*., 2018; Wu *et al*., 2018). The 1970s' Green Revolution marked a pivotal advancement in cereal production, driven by the integration of semi‐dwarfing genes with optimized fertilizer application and enhanced disease and insect resistance. These semi‐dwarf cultivars, widely adopted as Green Revolution varieties, have contributed to a steep increase in global grain yields over the past 6 decades (Evans and Lawson, 2020; Wan *et al*., 2021). With the ongoing rapid growth of the human population, maintaining stable per capita cereal availability now necessitates continuous expansion of total production to ensure global food security.

Both plant height and tiller number are critical determinants of rice plant architecture. Previous studies identified several genes that coordinately regulate these two traits. The *HIGH‐TILLERING DWARF 1* (*HTD1*) encodes the key enzymes involved in strigolactone (SL) biosynthesis that suppress the occurrence of tillers (Kulkarni *et al*., 2014). The *dwarf 10* (*d10*) mutant exhibits a characteristic multi‐tillered dwarf phenotype, featuring reduced plant height and increased tiller number. The *D10* gene encodes the carotenoid cleavage dioxygenase essential for strigolactone biosynthesis (Arite *et al*., 2007; Ito *et al*., 2010). Similarly, *HTD2*/*D14* encodes an esterase, with its mutants showing enhanced tillering and dwarfism (Arite *et al*., 2009; Liu *et al*., 2009). The D3 is assembled into a SCF^D3^ complex and combined with D14 to suppress aerial tiller development (Zhao *et al*., 2014). Additionally, the *Tillering and Dwarf 1* (*TAD1*)/*Tiller Enhancer* (*TE*) encodes a CDH1 homologue, which is an activation factor that promotes the APC/C complex through the antagonistic regulation of gibberellin (GA) and abscisic acid (ABA), thereby coordinating plant architecture (Lin *et al*., 2015; Xu *et al*., 2012). Furthermore, the *SMALL ORGAN SIZE 1* (*SMOS1*)/*REDUCED LEAF ANGLE 1* (*RLA1*)/*NITROGEN‐MEDIATED TILLER GROWTH RESPONSE 5* (*NGR5*) gene is induced by brassinosteroid (BR) and gibberellin (GA) and orchestrates the expression of genes associated with plant growth and development, thereby coordinating the regulation of plant height and tiller number in rice (Aya *et al*., 2014; Li *et al*., 2015; Qiao *et al*., 2017; Wu *et al*., 2020).

Gibberellin (GA), a diterpenoid acid that stimulates plant growth and is widely distributed in plant organs and tissues (Yamaguchi, 2008; Zhou *et al*., 2023), plays central roles in regulating plant architecture. The *semidwarf*‐*1* (*sd1*) gene, a cornerstone of the Green Revolution, encodes the GA20 oxidase critical for gibberellin biosynthesis. The discovery of *sd1* ushered in the era of semi‐dwarf varieties (Hedden, 2003; Sasaki *et al*., 2002; Spielmeyer *et al*., 2002). In the GA catabolic pathway, the GA2ox family members GA2ox6 and GA2ox9 both modulate plant height and tiller number (Lo *et al*., 2008; Xing *et al*., 2023). The *SLENDER RICE 1* (*SLR1*) encodes a DELLA protein that acts as a negative regulator of GA signalling. DELLA undergoes 26S proteasome‐mediated degradation to activate downstream GA‐responsive genes and regulates plant architecture (Gomi *et al*., 2004; Ikeda *et al*., 2001; Ueguchi‐Tanaka *et al*., 2007). Notably, SLR1 regulates plant height and tiller number in rice by inhibiting the degradation of MONOCULM1 (MOC1), which is the key regulatory factor for tiller development (Liao *et al*., 2019).

Previous studies have demonstrated that phosphoinositide (PI) metabolism plays a critical role in plant growth and development. As the most abundant phosphoinositides in cells, phosphatidylinositol 4‐phosphate (PI4P) and phosphatidylinositol 4,5‐bisphosphate [PI(4,5)P2] play essential roles in structural organization, intracellular trafficking and signal transduction (Marković and Jaillais, 2022; Nakamura, 2017; Wen *et al*., 2021). These anionic lipids likely interact directly with positively charged DNA‐binding proteins to regulate gene expression (Heilmann and Heilmann, 2024). In *Arabidopsis thaliana*, the *FRAGILE FIBER3* (*FRA3*) gene encodes a type II Inositol polyphosphate 5‐phosphatase (5PTase) that modulates PI metabolism, thereby influencing secondary wall biosynthesis and actin organization to shape plant developmental morphology (Zhong *et al*., 2004). In rice, the *DWARF 50* (*D50*) encodes a 5PTase required for internode elongation through actin organization (Sato‐izawa *et al*., 2012). Similarly, its homologous gene in maize, *BREVIS PLANT1* (*BV1*), implicates phosphoinositide signalling in stem elongation control (Avila *et al*., 2016). The *GRAIN NUMBER AND PLANT HEIGHT1* (*GH1*) gene in rice encodes a phosphatase containing a suppressor of actin (SAC) domain. Dysfunction of *GH1* results in PI(4,5)P2 overaccumulation, disrupting Arp2/3 complex‐mediated actin polymerization and impairing cellular development (Guo *et al*., 2019).

Rice plants carrying the *sd1* allele exhibit semi‐dwarfism accompanied by significantly enhanced lodging resistance and grain yield, yet reduced nitrogen use efficiency (NUE) (Wu *et al*., 2020). Thus, identifying and utilizing a novel semi‐dwarf allele will advance rice breeding programmes. In the present study, we identified a gene, *Moderate‐tillering and Semi‐dwarf 1* (*MTS1*), which encodes a type II 5PTase. The *mts1* mutant showed a significant reduction in plant height and an increase in effective tiller number compared to the wild type. The *MTS1* mutation impairs PI(4,5)P2 hydrolysis, resulting in its abnormal accumulation, which suppresses actin polymerization. We found that PI4P, the hydrolysis product of PI(4,5)P2, interacts with NGR5 and promotes NGR5 degradation, thereby influencing plant architecture. Collectively, these findings demonstrated that MTS1 regulates rice plant architecture by modulating phosphoinositide (PI) metabolism, which coordinates actin organization and affects NGR5 protein stability.

## Results

### Identification of a rice plant architecture mutant *mts1*

To identify novel genes regulating plant architecture in rice, we selected an *Oryza nivara* introgression line, Ra139 (referred to as the wild type [WT] hereafter), which exhibits a tall stem (167.8 ± 4.5 cm) and low tiller number (4.6 ± 0.8). This line carries the *SEMI‐DWARF 1* (*SD1*) allele derived from wild rice (Figure S1). Using ethyl methanesulfonate (EMS) mutagenesis, we generated a mutant population from Ra139. Through phenotypic screening of the mutant population grown in the field, we identified a mutant displaying semi‐dwarf characteristics, which we named *mts1* (*moderate‐tillering and semi‐dwarf 1*).

**Figure 1:**
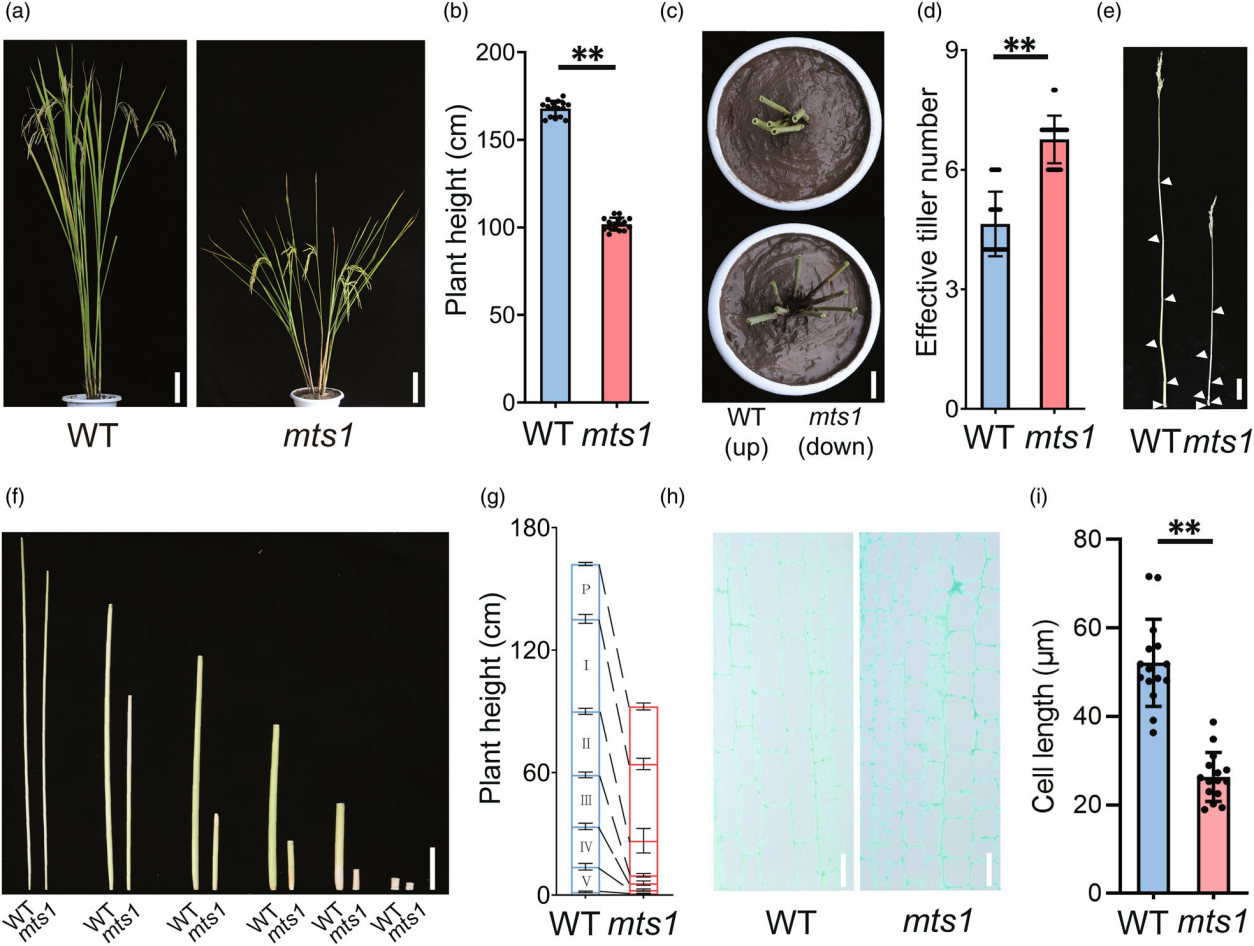
Phenotypic characterization of the *mts1* mutant. (a) Plant morphology of the wild type (Ra139) and the *mts1* mutant. Scale Bars, 15 cm. (b) Comparison of plant height in the wild type and the *mts1* mutant. Data represent mean ± SD (*n* = 16). Two‐tailed Student's *t*‐test (**P < 0.01). (c) Comparison of the base of the plant in the wild type and the *mts1* mutant. Vertical view. Scale bar, 5 cm. (d) Comparison of effective tiller number in the wild type and the *mts1* mutant. Data represent mean ± SD (*n* = 25). Two‐tailed Student's *t*‐test (**P < 0.01). (e–g) Comparison of internode length of main stem in the wild type and the *mts1* mutant. Internode designations: P (panicle), I (first internode), II (second internode), III (third internode), IV (fourth internode) and V (fifth internode). (e) Scale bar, 10 cm. (f) Scale bar, 5 cm. (g) Data represent mean ± SD (*n* = 5). (h) Longitudinal sections of stems from the wild type and the *mts1* mutant. Scale bars, 20 μm. (i) Comparison of cell length in stem of the wild type and the *mts1* mutant. Data represent mean ± SD (*n* = 15). Two‐tailed Student's *t*‐test (**P < 0.01).

Compared to the WT, the *mts1* mutant exhibited a significant reduction in plant height (101.9 ± 3.6 cm, a decrease of 39.2%) (Figure 1a,b) and an increase in effective tiller number (6.8 ± 0.6, an increase of 32.4%) (Figure 1c,d). Further analysis of the internode lengths of the main stems revealed that each internode was significantly shorter in the *mts1* mutant compared to the WT (Figure 1e–g). Histological examination of the fourth internode demonstrated that the *mts1* mutant had significantly shorter cell lengths than the WT (Figure 1h,i). Additionally, evaluation of other agronomically important traits showed that the *mts1* mutant had significantly shorter panicle length, fewer panicle branches and reduced grain number per panicle compared to the WT (Figure S2A–F). However, no significant differences were observed in grain length or width between the WT and the *mts1* mutant (Figure S2G,H,J,K). Notably, the *mts1* mutant maintained a similar grain yield per plant to the WT (Figure S2I,L). In summary, the mutation in the *MTS1* gene resulted in reduced plant height and increased effective tiller number without penalizing grain yield. These findings suggest that the *MTS1* allele could serve as a valuable genetic resource for plant architecture breeding in rice.

### Mapping and candidate gene identification of *MTS1*

**Figure 2:**
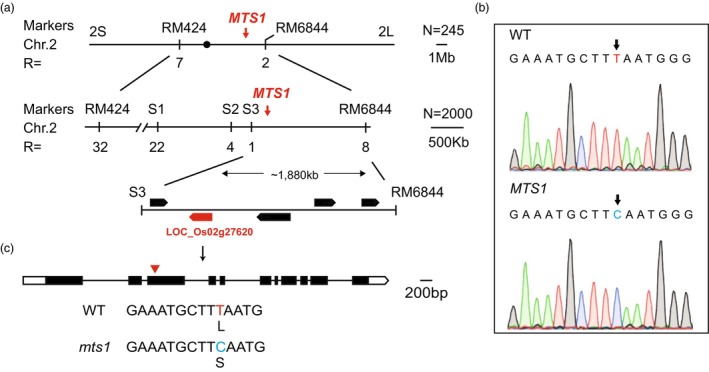
Mapping of *MTS1*. (a) Genetic mapping located *MTS1* between markers RM424 and RM6844 on chromosome 2 using 245 F_2_ individuals. The mapping location of the *MTS1* gene was further narrowed down to an approximately 1880 kb interval between marker S3 and RM6844 using 2000 F_2_ individuals. (b) The T‐to‐C point mutation in *LOC_Os02g27620*, detected using Sanger sequencing. (c) The exon‐intron structure of *LOC_Os02g27620*, black boxes represent exons, white boxes represent UTRs and thin black lines indicate introns. The T‐to‐C point mutation results in an amino acid change from leucine (L) to serine (S).

To analyse the genetic characterization of the phenotypes affected by the *MTS1* gene, we developed an F_2_ population from a cross between the wild type (WT) and the *mts1* mutant. Phenotypic analysis of 300 F_2_ individuals showed that there were 232 wild‐type plants and 68 mutant plants, fitting a 3:1 segregation ratio ($\chi^{2}$ = 0.75 < $\chi_{0.05,1}^{2}$ = 3.84). This indicates that the mutant phenotype in *mts1* is controlled by a single recessive gene. Using an F_2_ population derived from a cross between the *mts1* mutant and the *japonica* variety Zhonghua 17 (ZH17), we mapped the *MTS1* gene to a region between the SSR markers RM424 and RM6844 on the short arm of chromosome 2. To further fine mapping of *MTS1*, we developed a larger segregation population of 2000 individuals and narrowed down the gene into an approximately 1880 kb interval between molecular markers S3 and RM6844 (Figure 2a).

To identify the candidate gene for *MTS1*, we conducted whole‐genome resequencing to compare the genomic sequences of the WT and the *mts1* mutant. Within the fine‐mapped interval, we identified five single nucleotide polymorphisms (SNPs). Based on the annotation of the *japonica* variety *Nipponbare* reference genome, five predicted genes were located within 2 kb upstream or downstream of these SNPs. Notably, one SNP (T to C) in the third exon of *LOC_Os02g27620* (Figure 2b) resulted in an amino acid change from leucine (L) to serine (S) (Figure 2c; Figure S3), which alters the 3D structural conformation of the LOC_Os02g27620 protein in the WT and the *mts1* mutant (Figure S4). Previous studies have reported that *LOC_Os02g27620* corresponds to the *DWARF 50* (*D50*) gene, which regulates plant height in rice. These findings strongly suggest that *LOC_Os02g27620* is a candidate gene for *MTS1*.

### Isolation and characterization of *MTS1*

**Figure 3:**
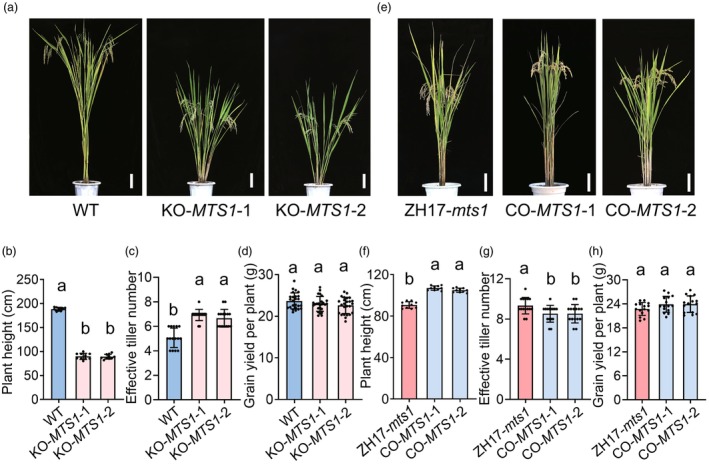
Functional verification of *MTS1*. (a) Plant morphology of the wild type (WT) and the knockout mutants (KO‐*MTS1*‐1 and KO‐*MTS1*‐2). Scale bars, 10 cm. (b–d) Comparison of plant height (b), effective tiller number (c) and grain yield per plant (d) in the wild type (WT) and the knockout mutants (KO‐*MTS1*‐1 and KO‐*MTS1*‐2). Data represent mean ± SD; sample size is 10 (b), 15 (c) and 25 (d), respectively. Different lowercase letters denote significant differences (ANOVA, *P* < 0.05). (e) Plant morphology of the ZH17‐*mts1* and the complementation transgenic lines (CO‐*MTS1*‐1 and CO‐*MTS1*‐2). Scale Bars, 10 cm. (f–h) Comparison of plant height (f), effective tiller number (g) and grain yield per plant (h) in the ZH17‐*mts1* and the complementation transgenic plants (CO‐*MTS1*‐1 and CO‐*MTS1*‐2). Data represent mean ± SD. Sample size is 10 (f), 15 (g) and 25 (h), respectively. Different lowercase letters denote significant differences (ANOVA, *P* < 0.05).

To confirm the function of *LOC_Os02g27620*, we developed a *MTS1* gene‐editing construct and introduced it into wild‐type (WT) plants (Figure S5A). The knockout mutants KO‐*MTS1*‐1 and KO‐*MTS1*‐2 harboured a 1‐bp deletion and a 50‐bp deletion, respectively, in the coding region of *LOC_Os02g27620*, resulting in frameshift mutations and premature translation termination. All homozygous KO‐*MTS1* mutant lines exhibited a significant reduction in plant height (89.6 ± 5.6 cm) compared to the WT (188.6 ± 3.5 cm) (Figure 3a,b). Histological analysis of the fourth internode revealed that cell length in KO‐*MTS1* plants was significantly shorter than in the WT (Figure S5B–D), while the effective tiller number was significantly higher in KO‐*MTS1* than in the WT (Figure 3c). Although several yield‐related traits differed significantly between the WT and KO‐*MTS1*, no significant difference was observed in grain yield per plant (Figure 3d; Figure S5E–O). Additionally, we generated an RNA interference (RNAi) construct targeting *LOC_Os02g27620* and introduced it into the *japonica* variety Zhonghua 17 (ZH17). Phenotypic analysis showed that RNAi transgenic plants (RNAi‐*MTS1*) exhibited a significant decrease in plant height and an increase in effective tiller number compared to the control (ZH17) (Figure S6A–D). These results from the genome editing and RNAi experiments demonstrate that *LOC_Os02g27620* plays a critical role in regulating plant architecture in rice.

To investigate whether the SNP in the coding region of *LOC_Os02g27620* was responsible for the altered plant architecture in the *mts1* mutant, we conducted a genetic complementation test. Due to the difficulty in regenerating shoots from callus tissue of the *mts1* mutant, we developed a near‐isogenic line, ZH17‐*mts1*, which carries the *mts1* mutation allele in the *japonica* variety ZH17 background. A complementation construct (CO‐*MTS1*), containing a 6684‐bp wild‐type genomic fragment encompassing the entire coding sequence, 2598‐bp of the 5′‐flanking region and 466‐bp of the 3′‐flanking region, was introduced into ZH17‐*mts1* via Agrobacterium‐mediated transformation. Ten independent positive transgenic lines (CO‐*MTS1*) were generated, all of which showed a significant increase in plant height and a decrease in effective tiller number compared to the control (ZH17‐*mts1*) (Figure 3e–g). Histological analysis of the fourth internode revealed that cell length was significantly longer in CO‐*MTS1* than in ZH17‐*mts1* (Figure S7A–C). Notably, CO‐*MTS1* exhibited a similar grain yield per plant to ZH17‐*mts1*, despite significant differences in other yield‐related traits (Figure 3h; Figure S7D–M).

In summary, the genetic complementation test confirmed that the T‐to‐C point mutation in the third exon of *LOC_Os02g27620* is responsible for the reduced plant height and increased effective tiller number, resulting in a semi‐dwarf phenotype. These findings demonstrate that *LOC_Os02g27620* is equivalent to the *MTS1* gene.

### Expression pattern and molecular function of *MTS1*

The open reading frame (ORF) of MTS1 is 3243 bp in length and encodes a protein of 1080 amino acid residues. The protein contains a WD40 repeat domain (residues 428–544) and an EEP domain (residues 566–903) (Figure S3). Analysis of the predicted amino acid sequence suggests that *MTS1* encodes a putative 5PTase with phosphohydrolase activity. Phylogenetic analysis revealed that the MTS1 protein was evolutionarily most closely related to orthologs in *Brachypodium distachyon* (Bradi3g43457) (Figure S8), and it is homologous to *BV1* in *Zea mays* and *FRA3* in *Arabidopsis thaliana* (Figure S9). Further measurement of PI4P concentration revealed that both the *mts1* mutant and the Cas9‐*MTS1* plants had significantly lower PI4P levels compared to the wild type (WT), confirming that *MTS1* hydrolyzes PI(4,5)P2 to PI4P (Figure S10).

**Figure 4:**
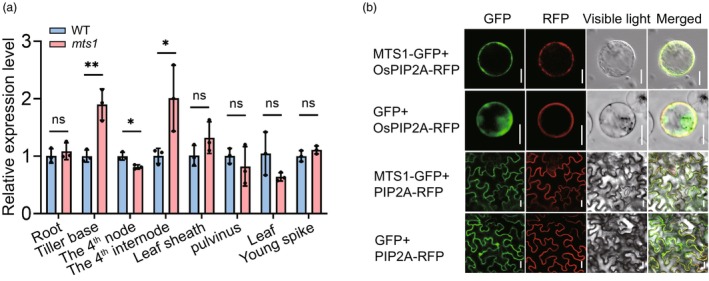
The expression pattern of *MTS1*. (a) Comparison of expression levels of *MTS1* between the wild‐type (WT) and the *mts1* mutant. Data represent means ± SD (*n* = 3). Two‐tailed Student's *t*‐test (**P < 0.01, *P < 0.05, ns, not significant). (b) Subcellular localization of MTS1 in the rice protoplasts (Scale bar, 10 μm) and *Nicotiana benthamiana* leaves (Scale bar, 25 μm). The PIP2A‐RFP fusion protein was used as a plasma membrane marker.

To investigate the temporal and spatial expression patterns of *MTS1*, we performed reverse transcription quantitative PCR (RT‐qPCR) analysis. The results showed that *MTS1* is expressed in all examined tissues, with particularly high expression levels in the tiller base and the fourth internode (Figure 4a). This expression pattern is consistent with the observed phenotypic effects of *MTS1* on plant architecture. To determine the subcellular localization of the MTS1 protein, we developed a MTS1‐GFP fusion construct and co‐transformed it into rice protoplasts and *Nicotiana benthamiana* leaves alongside a PIP2A‐RFP fusion construct, as a plasma membrane marker. Both GFP and RFP fluorescence signals were observed at the cell membrane, indicating that MTS1 is primarily localized to the plasma membrane (Figure 4b).

### *MTS1* mutation disrupts actin cytoskeletal organization

**Figure 5:**
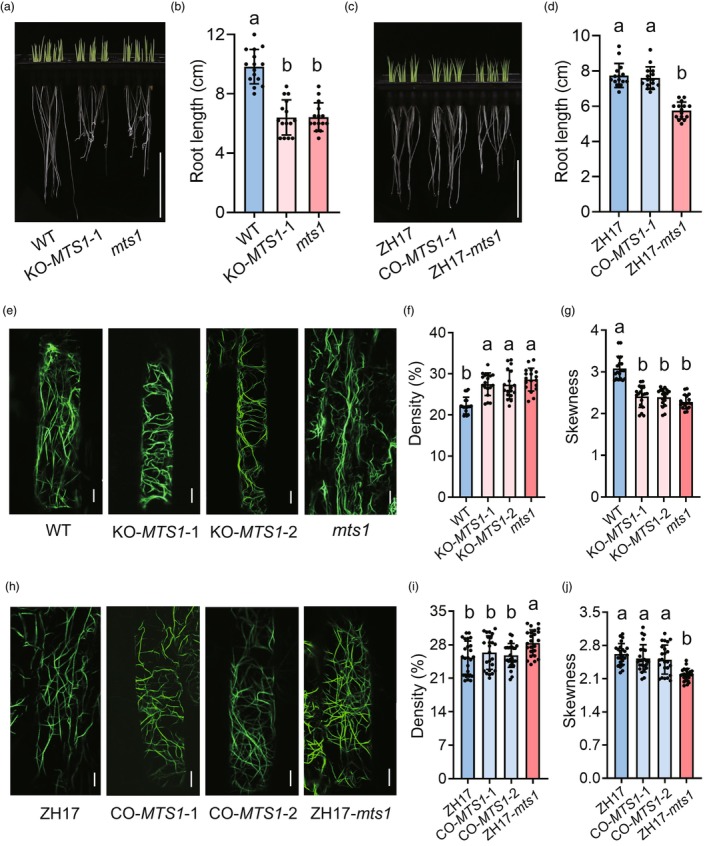
Morphology of root microfilaments. (a) Rice seedlings of the wild type (WT), knockout mutant (KO‐*MTS1*‐1) and the *mts1* mutant. Scale Bar, 5 cm. (b) Comparison of root length among the wild type (WT), knockout mutant (KO‐*MTS1*‐1) and the *mts1* mutant. Data represent means ± SD (*n* = 15). Different lowercase letters denote significant differences (ANOVA, *P* < 0.05). (c) Rice seedlings of *japonica* variety ZH17, the complementation transgenic line (CO‐*MTS1*‐1) and ZH17‐*mts1*. Scale bar, 5 cm. (d) Comparison of root length among *japonica* variety ZH17, the complementation transgenic line (CO‐*MTS1*‐1) and ZH17‐*mts1*. Data represent means ± SD (*n* = 15). Different lowercase letters denote significant differences (ANOVA, *P* < 0.05). (e) Morphology of root microfilaments of the wild type (WT), knockout mutant (KO‐*MTS1*‐1 and KO‐*MTS1*‐2) and the *mts1* mutant. Scale bars, 5 μm. (f, g) Comparison of the density (F) and skewness (G) of microfilaments among the wild type (WT), knockout mutant (KO‐*MTS1*‐1 and KO‐*MTS1*‐2) and the *mts1* mutant. Data represent means ± SD (*n* = 18). Different lowercase letters denote significant differences (ANOVA, *P* < 0.05). (h) Morphology of root microfilaments of *japonica* variety ZH17, the complementation transgenic line (CO‐*MTS1*‐1 and CO‐*MTS1*‐2) and ZH17‐*mts1*. Scale bars, 5 μm. (i, j) Comparison of the density (i) and skewness (j) of microfilaments among the *japonica* variety ZH17, the complementation transgenic line (CO‐*MTS1*‐1 and CO‐*MTS1*‐2) and ZH17‐*mts1*. Data represent means ± SD (*n* = 18). Different lowercase letters denote significant differences (ANOVA, *P* < 0.05).

Actin, a core component of the cytoskeleton, plays a critical role in regulating plant development. Dysfunction of SAC phosphatase can lead to the overaccumulation of phosphatidylinositol 4,5‐bisphosphate [PI(4,5)P2], which disrupts actin polymerization and consequently results in abnormal plant development (Guo *et al*., 2019). To determine whether the mutation of *MTS1* affects actin cytoskeleton organization in roots, we first measured root length at 4 days after germination. Both *MTS1* mutant and KO‐*MTS1* plants exhibited significantly shorter roots compared to the wild type (WT), while CO‐*MTS1* and control (ZH17) plants exhibited longer roots compared to ZH17‐*mts1* (Figure 5a–d).

To visualize actin organization, we stained root elongation zones with ActinGreen™ 488. Actin polymerization was evaluated via both density and skewness of microfilament as metrics (Higaki *et al*., 2010; Li *et al*., 2023). Confocal laser scanning microscopy revealed that WT roots showed lower microfilament density and higher skewness than the *mts1* mutants and KO‐*MTS1* plants (Figure 5e–g). Similarly, CO‐*MTS1* and control (ZH17) plants also exhibited lower microfilament density and higher skewness than ZH17‐*mts1* plants (Figure 5h–j). These findings indicated that the mutation of *MTS1* impairs the hydrolysis of PI(4,5)P2, resulting in its aberrant accumulation and subsequent suppression of actin polymerization.

### Mutation of *MTS1* specifically affects GA response at the reproductive growth stage

**Figure 6:**
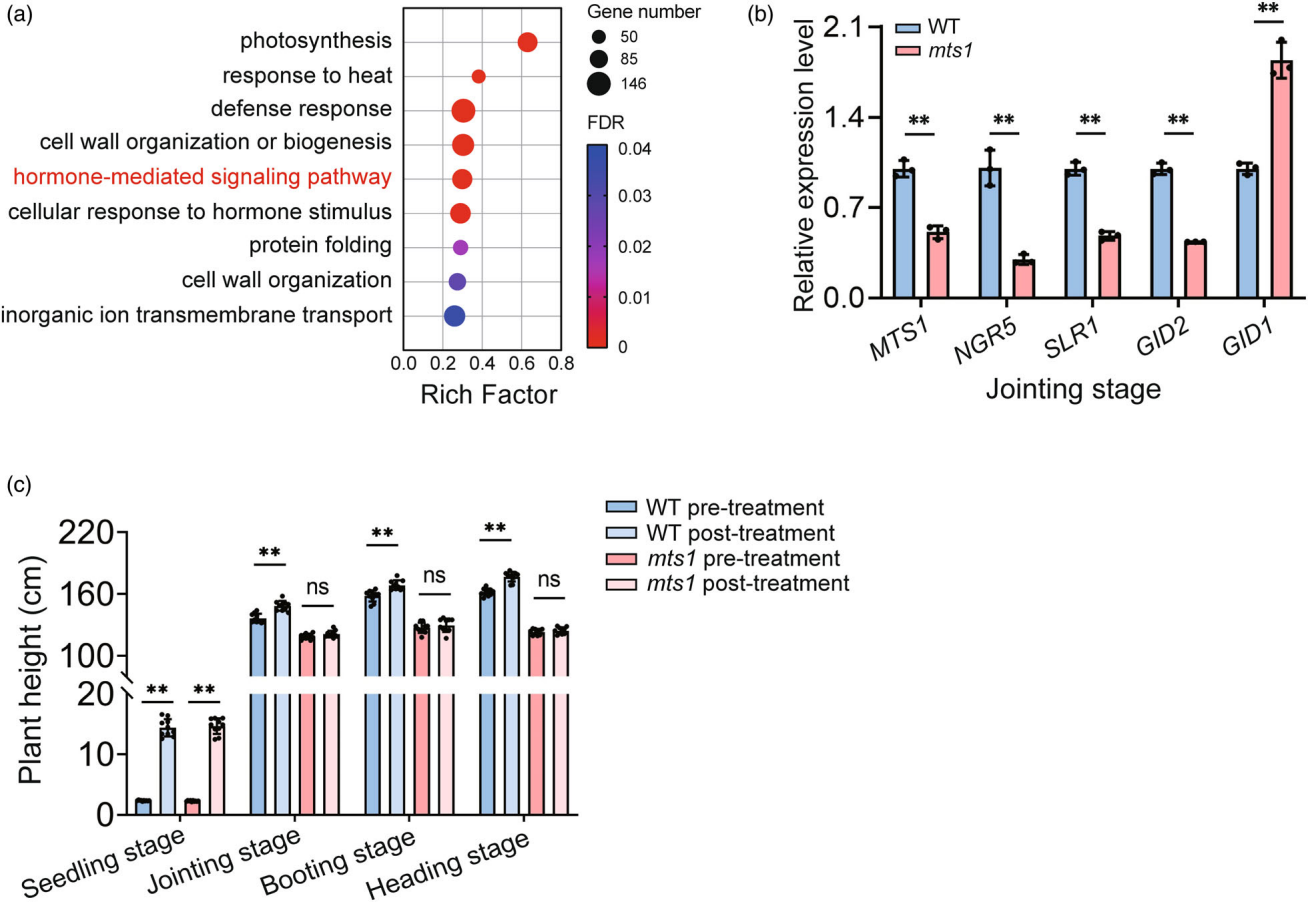
The mutation of *MTS1* affected gibberellin response. (a) GO analysis of differentially expressed genes between the wild type and the *mts1* mutant. (b) Comparison of expression levels of *MTS1* and GA signal transduction genes in the fourth internode of the wild‐type (WT) and the *mts1* mutant at the jointing stage. Data represent means ± SD (*n* = 3). Two‐tailed Student's *t*‐test (**P < 0.01). (c) Comparison of plant height in the wild type and the *mts1* mutant before and after GA treatment (30 ppm GA_3_) at the seedling, jointing, booting and heading stage. Data represent means ± SD (*n* = 10). Two‐tailed Student's *t*‐test (**P < 0.01, ns, not significant).

To further explore the molecular mechanisms by which *MTS1* regulates plant architecture, we conducted RNA‐sequencing (RNA‐seq) analysis. A total of 6172 differentially expressed genes (DEGs) were identified in the fourth internode between the WT and the *mts1* mutant, including 3292 up‐regulated and 2880 down‐regulated genes (fold change ≥2, FDR < 0.0001). Gene Ontology (GO) enrichment analysis revealed that these DEGs are involved in multiple biological processes, including response to gibberellin, small molecule metabolic processes, cell wall macromolecule metabolic processes and regulation of transmembrane transport (Figure 6a).

In general, gibberellins (GAs) play a pivotal role in regulating plant height by influencing both cell elongation and cell division. GO enrichment analysis of differentially expressed genes (DEGs) between wild‐type (WT) and the *mts1* mutant plants revealed that the mutation in *MTS1* might alter the response to gibberellin. Additionally, RT‐qPCR analysis showed that there were significant differences in the expression levels of several important GA signal‐related genes in the fourth internode of both WT and *mts1* mutant plants (Figure 6b). To validate whether the *MTS1* gene modulates plant architecture through its response to gibberellin, experiments were conducted to measure the plant height of both WT and *mts1* mutant plants treated with GA_3_ at both vegetative and reproductive growth stages. The results showed that, in WT plants, the application of GA_3_ significantly increased plant height at both growth stages. However, in *mts1* mutant plants, no obvious difference was observed before and after GA_3_ treatment at the jointing, booting and heading stages, while GA_3_ treatment resulted in a significant increase in plant height at the seedling stage (Figure 6c). Furthermore, additional experiments confirmed that the plant height of *mts1* mutant plants remained unchanged regardless of the GA_3_ concentration (10, 20, 30 ppm) used during the reproductive growth stage (Figure S11). Taken together, these findings strongly suggest that the mutation in *MTS1* modulates rice plant height by specifically affecting the response to gibberellin at the reproductive growth stage.

### The hydrolysis product PI4P of *MTS1* can promote the degradation of NGR5

**Figure 7:**
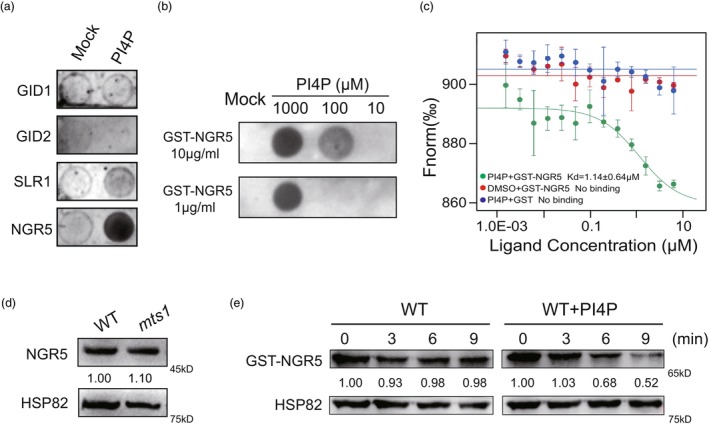
PI4P interacts with NGR5 and promotes NGR5 degradation. (a) Detection of interactions between PI4P and GA transduction‐related genes. (b) Detection of interaction between PI4P and NGR5 in different concentrations of PI4P and GST‐NGR5 recombinant protein. (c) The binding affinity of NGR5 to PI4P in the MST assay. NHS‐labelled GST‐NGR5 recombinant protein is titrated to a varying concentration of PI4P. Error bars represent SD (*n* = 3). (d) Immunoblot analysis of NGR5 in the wild type (WT) and the *mts1* mutant. The rice housekeeping protein HSP82 served as the loading control. (e) Investigation of GST‐NGR5 recombinant protein stability before and after the introduction of PI4P into total proteins extracted from seedlings of wild type.

Previous studies had demonstrated that phospholipid small molecules (PIs) can interact with proteins and influence the stability of their interacting partners (Boss and Im, 2012; Vidalle *et al*., 2023). To explore whether changes in PI4P concentration affect plant architecture development by binding to proteins associated with the GA signalling pathway, we conducted a lipid–protein overlay assay to examine the interaction between PI4P and four GA signalling‐related proteins, including GID1, GID2, SLR1 and NGR5. Our results revealed that PI4P specifically interacts with NGR5 (Figure 7a,b), which is known to play a critical role in regulating plant architecture in rice (Qiao *et al*., 2017). Microscale thermophoresis (MST) assays further confirmed the interaction between PI4P and NGR5, showing a sigmoid curve pattern with a dissociation constant (Kd) of 1.14 ± 0.6 μM for the PI4P‐NGR5 binding (Figure 7c). In contrast, no binding was detected in the two control groups (DMSO with NGR5 and PI4P with GST). To determine whether PI4P binding affects the stability of NGR5, we performed a cell‐free degradation assay. The addition of PI4P to the extracts promoted the degradation of NGR5 compared to the control. Immunoblot analysis further revealed that the expression level of NGR5 was significantly higher in the *mts1* mutant than in the WT (Figure 7d). The detection of the NGR5 protein antibody has specificity (Figure S12), so we incorporated PI4P into the WT system and cell‐free experiments revealed the introduction of PI4P into the system markedly enhanced NGR5 degradation (Figure 7e). These findings collectively indicated that PI4P interacts with NGR5 and promotes its degradation, thereby modulating plant architecture in rice.

### Evaluation of the breeding potential of *MTS1* gene in rice

**Figure 8:**
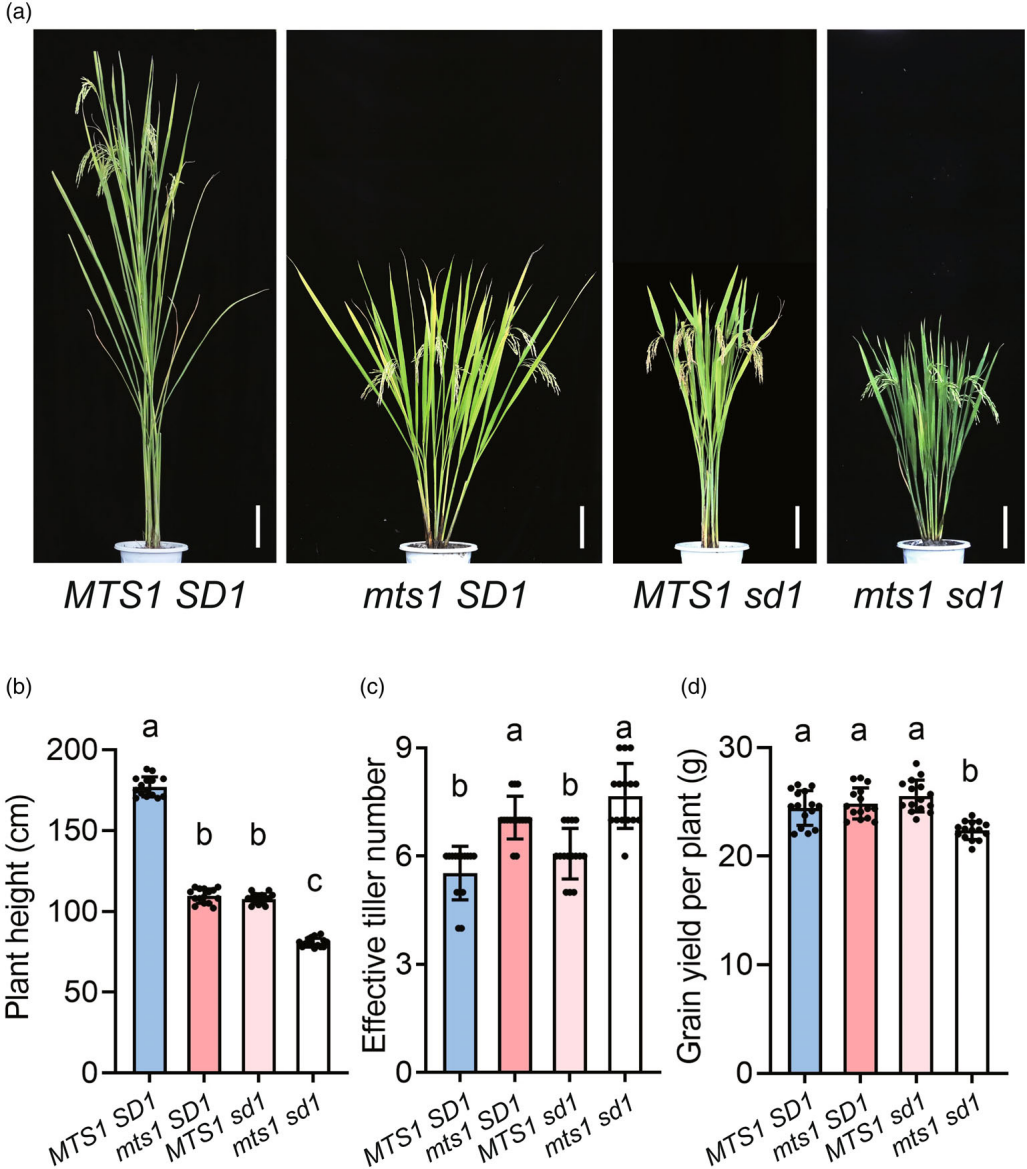
Evaluation of the potential of *MTS1* in rice breeding. (a) Plant morphology of four near‐isogenic lines (NILs) carrying allelic combinations of *MTS1*/*SD1*. Scale bars, 15 cm. (b–d) Comparison of plant height (b), effective tiller number (c) and grain yield per plant (d) among four near‐isogenic lines (NILs) carrying allelic combinations of *MTS1*/*SD1*. Data represent means ± SD (*n* = 15). Different lowercase letters denote significant differences (ANOVA, *P* < 0.05).

Improving plant architecture is critical for enhancing grain yield in rice. It is well known that the application of the semi‐dwarf allele *sd1* drove the historic ‘green revolution’ by reducing lodging and increasing harvest index. To evaluate the breeding potential of the *MTS1* gene, we developed four near‐isogenic lines (NILs) carrying allelic combinations of *MTS1*/*SD1*: *MTS1 SD1*, *mts1 SD1*, *MTS1 sd1* and *mts1 sd1* (Figure 8a). Phenotypic analysis revealed that the plant height of both NILs *mts1 SD1* and *MTS1 sd1* was higher than that of the NIL *mts1 sd1* (Figure 8b), suggesting that *MTS1* and *SD1* may exhibit additive effects in plant height. Notably, NIL *mts1 SD1* had a similar effective tiller number to NIL *mts1 sd1* and NIL *MTS1 SD1* had a similar effective tiller number to NIL *MTS1 sd1*. However, both *mts1 SD1* and *mts1 sd1* had a greater effective tiller number than *MTS1 SD1* and *MTS1 sd1*, demonstrating that the mutation of *MTS1* can increase effective tiller number (Figure 8c). Further phenotype analysis showed that, although there were differences in yield‐related traits (Figure S13A–H), there was no significant difference in grain yield per plant between *mts1 SD1* and *MTS1 sd1* (Figure 8d), attributed to the compensatory effect of effective tiller number. Therefore, we speculated that *MTS1* possesses breeding potential comparable to that of *SD1*. These results suggest that the *mts1* mutant may have some negative effects on agronomic traits, but its grain yield per plant is not affected due to the compensation effect of increasing effective tiller number. Therefore, *mts1* has potential application value in rice dwarf breeding; it provides a novel genetic basis for optimizing plant architecture without compromising productivity.

### The *MTS1* locus underwent selection during *japonica* rice domestication

**Figure 9:**
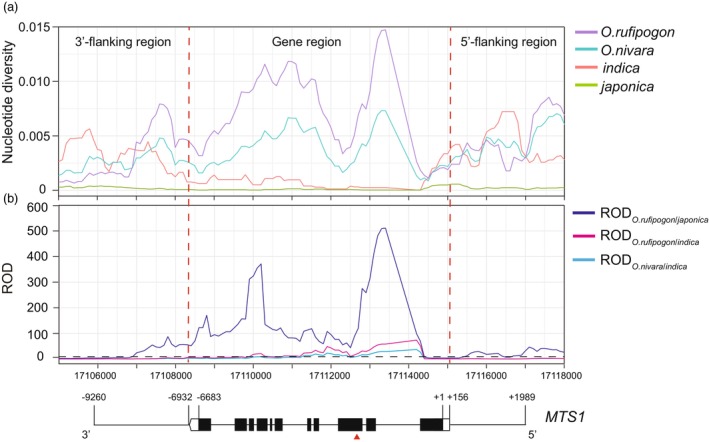
Nucleotide diversity and reduction of diversity (ROD) of the *MTS1* locus in wild and cultivated rice. (a) Sliding‐window analysis of nucleotide polymorphism (*π*) in *MTS1*. The values were calculated for each sliding window of 1 kb with an increment of 100 bp. (b) Reduction of diversity (ROD) analysis. Black dotted line represents the threshold value (2.615).

Given that the *MTS1* mutation confers improved plant architecture, we investigated its evolutionary trajectory by analysing natural variation in *MTS1* genomic sequences (including the 2145 bp 5′‐flanking region, 6683 bp coding region and 2577 bp 3′‐flanking region) across 134 *O. rufipogon*, 157 *O. nivara*, 301 *japonica* and 575 *indica* accessions (Jing *et al*., 2023). The results showed that the *MTS1* gene region exhibited higher nucleotide diversity (*π*) than its 5′‐ and 3′‐flanking regions in wild and cultivated rice. Notably, wild rice (*O. rufipogon* and *O. nivara*) had significantly higher nucleotide diversity in the *MTS1* gene region than cultivated rice (*indica* and *japonica*), while the nucleotide diversity had no obvious difference in the 5′‐ and 3′‐flanking regions between wild and cultivated rice (Figure 9a). To further quantify selection pressure, we calculated the reduction of diversity (ROD) which is the ratio of nucleotide polymorphism (π) between two groups. The results showed that the ROD between *O. rufipogon* and *japonica* in the *MTS1* gene region was obviously higher than that both between *O. rufipogon* and *indica* and between *O. nivara* and *japonica*, and also higher than the genome‐wide ROD threshold of 2.615 (Figure 9b). A total of 1167 accessions were further classified into 10 haplotypes using the 26 variations (minimum allele frequency >3%) in the *MTS1* gene region. 96.0% of *indica* carried Hap1 and Hap2 and 90.0% of *japonica* carried Hap3 (Figure S14). These results conclusively demonstrated that the *MTS1* locus underwent strong directional selection during *japonica* domestication and *MTS1* exhibits the differentiation between *indica* and *japonica* subspecies.

## Discussion

Both plant height and effective tiller number are pivotal traits for achieving ideal plant architecture in rice (Fan *et al*., 2024). The domestication and optimization of plant architecture have historically driven rice productivity by maximizing population‐level yield through enhancing lodging resistance and population photosynthetic efficiency. Therefore, the development of rice varieties with ideal plant architecture remains a central objective in modern breeding programmes. However, the majority of current rice varieties carry the Green Revolution gene *sd1*, the singular source of the semi‐dwarfism gene, which has severely limited the development of new rice varieties. Although numerous dwarf mutants have been identified in rice, most of these dwarf mutants often exhibit extreme dwarfism, poor fertility and low grain yield, rendering these dwarfing gene resources largely unsuitable for utilization in rice breeding programmes. To address this limitation, we screened a rice mutagenized population and identified the *mts1* mutant, which exhibits semi‐dwarf morphology without compromising grain yield. Notably, compared to the near‐isogenic line carrying *MTS1* and *SD1* alleles, the near‐isogenic line carrying *mts1* and *SD1* alleles has a significant decrease in plant height and a similar grain productivity, implying that the *mts1* allele has a semi‐dwarfism ability comparable to *sd1*. Therefore, the *mts1* allele has a new semi‐dwarfism gene resource and significant potential for rice breeding applications.

Phosphoinositide (PI) metabolism involves structural organization, intracellular trafficking and signal transduction, thereby modulating plant growth and development. PIs control organelle biology by regulating vesicular trafficking, but they also modulate lipid distribution and metabolism via their close relationship with lipid transfer proteins. The *Arabidopsis FRAGILE FIBER 3* (*FRA3*) gene encodes a type II inositol polyphosphate 5‐phosphatase (5PTase) that hydrolyses PI(4,5)P2 to PI4P. The mutation of *FAR3* results in abnormal plant morphology due to the change in actin cytoskeleton organization and secondary wall biosynthesis (Zhong *et al*., 2004). The rice *DWARF 50* (*D50*) gene, a homologous gene of *FRA3*, is involved in modulating the development of intercalary meristem. The mutation of *D50* results in a dwarf phenotype due to abnormally organized cell files. In the *d50* mutant, a G‐to‐A mutation at nucleotide position 1023 of the coding region results in the conversion of tryptophan (W) to introduce a premature termination codon (Sato‐Izawa *et al*., 2012). Notably, the *mts1* allele is a new mutation allele of *D50*. Our findings demonstrated that the mutation of *MTS1* affects the activation of 5PTase to change the actin microfilament structure, resulting in reduced plant height and increased tiller number.

Interestingly, previous studies discovered that PIs are present not only in the plasma membrane but also in the nucleus. Inositol phospholipids, inositol phosphates and enzymes of the phosphoinositide pathway in plant nuclei are involved in DNA replication, chromatin remodelling, stress responses and hormone signalling (Champeyroux *et al*., 2020). Several studies reported that the GA treatment in *Arabidopsis* could enhance nucleoplasmic phospholipid small molecules, indicating that nuclear PIs may belong to a new regulatory loop initiated by GA and PIs that was utilized for signalling within the nucleus (Dieck *et al*., 2012; Minasbekyan *et al*., 2008). In the present study, we found that the mutation in *MTS1* modulates rice plant height by specifically affecting the response to gibberellin at the reproductive growth stage, and PI4P, the downstream hydrolysis product of type II inositol polyphosphate 5‐phosphatase (5PTase), could interact with NGR5, a key regulatory factor in the GA signalling pathway. Meanwhile, the mutation of *MTS1* results in reducing PI4P concentration, enhancing the accumulation of NGR5, thereby changing rice plant architecture. However, the molecular mechanism underlying how GAs and PIs signalling pathways coordinately regulate rice plant architecture remains to be further elucidated in the future.

Brassinoteroids (BRs) critically regulate rice plant architecture (Yang *et al*., 2024). In rice, *GSK3/SHAGGY‐like kinase 2* (*GSK2*) suppresses the expression of downstream BR‐responsive genes, whereas DWARF AND LOW‐TILLERING (DLT) is a positive regulator that mediates BR response. GSK2 modulates plant architecture by phosphorylating DLT and SMOS1/RLA1/NGR5, which act as integrators of the transcriptional complex and play an essential role in BR signalling and plant height in rice (Hirano *et al*., 2017; Qiao *et al*., 2017; Tong *et al*., 2012). Notably, a total of 24 BR signalling‐related genes were differentially expressed in the WT and the *mts1* mutant (Table S1), suggesting that the mutation of *MTS1* results in enhanced accumulation of NGR5, thereby involving itself in the BR signalling pathway to regulate plant architecture in rice. Therefore, further studies are needed to elucidate how the *MTS1* gene regulates rice plant architecture via BR signalling.

During rice domestication, favourable variations were selected and accumulated, continuously enhancing grain yield, improving grain quality, strengthening stress resistance and increasing adaptability. In the present study, we found that the *mts1* allele could be utilized for improving plant architecture. Concurrently, molecular evolutionary analysis revealed that the *MTS1* locus underwent strong selection during *japonica* rice domestication, suggesting that its favourable natural variation might have also contributed to plant architecture improvement in *japonica* rice. However, the key functional mutations underlying plant architecture improvement remain to be fully characterized. Current studies showed that diverse genetic variations could be developed using genome editing technology. Considering the application potential of the rice *MTS1* gene, applying CRISPR/Cas9 to edit the *MTS1* gene could create novel superior allelic variants of *MTS1*, which would provide new genetic resources for breeding new varieties with ideal plant architecture in the future.

## Materials and methods

### Plant materials and phenotypic evaluation

The wild type (Ra139) was an introgression line derived from a backcross between *O. nivara* (W2014), as the donor parent and *indica* variety 9311, as the recipient parent. The *mts1* were identified from the mutagenized population of Ra139. An F_2_ population was derived from a cross between the mts1 mutant and the *japonica* variety Zhonghua 17 (ZH17). Rice seedlings were cultivated in a greenhouse under 40% humidity at 28°C conditions at China Agricultural University. All plant materials were grown in the field at the experimental stations of China Agricultural University, located in Beijing and Hainan Province, China. The agronomical traits, including plant height, effective tiller number, panicle length, grain number per panicle, grain length, grain width, grain weight and grain yield per plant, were measured.

### Primers

The primers used in the present study are listed in Table S2.

### Constructs development and transformation

Using wild‐type genome DNA as a template, approximately 8 kb around the *MTS1* gene was amplified and inserted into the binary vector pCAMBIA1300 by the recombinant enzyme system (Seamless Assembly Cloning Kit, CloneSmarter) to form the genomic function complementation construct. Using wild‐type cDNA as a template, an inverted repeat harbouring approximately 300–400 bp was amplified and inserted into the pTCK303/JL1460 vector to form the RNAi construct. The editing of *MTS1* to generate knockout mutants was based on CRISPR‐Cas9 technology (Ma *et al*., 2015). All vectors were introduced into the EHA105 competent state of *Agrobacterium tumefaciens*, followed by *Agrobacterium*‐mediated transformation into the recipient materials.

### Reverse transcription quantitative PCR

Total RNAs were extracted from various rice tissues using TRizol® (Invitrogen, USA) following the manufacturer's instructions. Reverse transcription quantitative PCR (RT‐qPCR) was performed using the StepOnePlus Real Time PCR System (Applied Biosystems, Thermo Fisher Scientific, Beijing, China). The rice *Ubiquitin* gene was used as an internal control. The 2^−ΔΔCT^ method was employed to calculate the relative expression, with each set of experiments being repeated three times.

### RNA‐sequencing data analysis

Total RNAs were extracted from the internode tissues of the wild type and the *mts1* mutant at the jointing stage and used to analyse genome‐wide expression pattern using the Illumina HiSeq2500 sequencing platform (Novogene, Beijing, China). Each set of experiments had three biological replicates. Clean data were aligned to the reference genome (Os‐Nipponbare‐Reference‐IRGSP‐1.0, MSU7) using Tophat2 software (Kim *et al*., 2013). Differentially expressed genes (fold change ≥2 and FDR <0.0001) were identified between the wild type and the *mts1* mutant using DESeq2. Subsequently, the functional analysis of the differentially expressed genes were performed using AgriGO (Tian *et al*., 2017).

### Subcellular localization assays

The construct p*35S*:*OsPIP2*‐*RFP* was used as a cytomembrane localization marker. Both p35S:*MTS1*‐*GFP* and p*35S*:*OsPIP2*‐*RFP* constructs were co‐introduced into rice protoplasts and incubated for 16 h at 28 °C in the dark. The GFP and RFP fluorescence was visualized with 488 nm and 543 nm laser lines using LSM 880 confocal laser scanning microscope (Zeiss, Shanghai, China). Additionally, the p35S:*MTS1*‐*GFP* and p*35S*:*OsPIP2*‐*RFP* vectors were introduced into *A. tumefaciens* GV3101 and then infiltrated into *N. benthamiana* leaves. The fluorescence was observed 3 days after infiltration.

### GA sensitivity analysis

The seedlings at the one leaf stage were treated with 1 μL of 1 × 10^−5^ M GA_3_ on the coleoptile. The GA_3_‐treated seedlings were then cultured at 28 °C for 10 days under a 12‐h light and 12‐h dark cycle, and the length of the second leaf sheath was subsequently measured. Additionally, 20 plants of the wild type and the *mts1* mutant were randomly chosen at the jointing and booting stages, and the height of each plant was measured. These selected plants were sprayed with GA_3_ at varying concentrations (0, 10, 20, 30 ppm), with 100 mL per plant for 10 consecutive days. Following the treatment period, the height of each plant was measured again.

### Histological analysis

The fourth internodes of the wild‐type and the *mts1* mutant were embedded in paraffin. Paraffin blocks were sectioned with a LEICA RM2265 microtome at a thickness of 8–10 mm, and subsequently stained before being observed under an Olympus BX51 microscope.

### Microfilament staining and visualization

To visualize the actin cytoskeleton in rice cells, root tips of 4‐day‐old rice seedlings were treated with PEM buffer reagent containing ActinGreen488 for 30 min at room temperature. Following this treatment, the actin filaments were rinsed with PBS and examined using Airyscan technology on the LSM 880/900 confocal laser scanning microscope (Zeiss) with excitation at 488 nm. The resulting images were analysed with ImageJ software.

### Protein expression and purification

Recombinant GST‐NGR5, GST‐SLR1, His‐GID1 and GST‐GID2 proteins were expressed in M5 HiPer Rosetta (DE3) competent cells and purified using glutathione sepharose and Ni sepharose columns.

### Lipid–protein overlay assay

The PVDF membrane was spotted with 1–2 μL of solvent and allowed to dry for 30 min. Then, the PVDF membrane was incubated for 1–3 h. The PVDF membrane was incubated for 1 h with GST‐tagged proteins in blocking solution. The binding proteins were removed from the blocking solution and washed twice with 5% (w/v) PBST. Then, the binding proteins were detected using anti‐GST antibodies (EasyBio, Beijing, China) as described previously (Yang *et al*., 2021).

### *In vivo* cell‐free degradation assays

The cell‐free assay was performed as described by Hao *et al*. (2023). The 15‐day‐old seedlings of the WT and the *mts1* mutant were ground, and total proteins were subsequently extracted in a degradation buffer containing 50 mM Tris–HCl (pH = 8.0), 0.5 M sucrose, 1 mM MgCl_2_, 10 mM EDTA (pH = 8.0), 5 mM DTT and 100 mM ATP. The 300 ng recombinant GST‐NGR5 proteins were incubated in 200 μL extracts (containing 1400 μg total proteins) for the individual assays per reaction. Proteins were incubated for 0, 3, 6 and 9 min at 23 °C, and immunoblots detected samples using anti‐GST antibody (EasyBio, Beijing, China). Rice HSP82 protein was used as a loading control (Huang *et al*., 2018). The results were quantified using ImageJ software.

### Microscale thermophoresis assay

The Microscale thermophoresis (MST) assay was conducted using a Nano Temper Monolith (NT.115) instrument. Recombinant GST‐NGR5 was labelled with NHS NT‐647 dye. Then, the labelled peptides were diluted to a concentration of 10 nM in phosphate buffer containing 0.005% Tween‐20. The peptides labelled with NT‐647 dye and PI4P were then combined in equal proportions. Following a 30‐min incubation period in the dark, the mixture was loaded into capillaries (Nano Temper Technologies, Munich, Germany) for analysis. The experiments were carried out using 20% LED and 20% MST power. The dissociation constant (KD) was determined based on the thermophoresis C T‐Jump results.

### Homology analysis

The homologous proteins were identified from the phytozome database (https://phytozome.jgi.doe.gov/pz/portal.html). An amino acid multiple sequence alignment was performed using ClustalX 1.83. A phylogenetic tree was constructed using MEGA 6.0 (neighbour‐joining) with 1000 bootstrap repetitions (Tamura *et al*., 2013).

### Nucleotide diversity analysis

A previously published dataset of 1167 diverse rice accessions (134 *O. rufipogon*, 157 *O. nivara*, 301 *japonica* and 575 *indica*) (Jing *et al*., 2023) was used to calculate the nucleotide diversity (π) using VCFtools. The sliding window was1 kb with an increment of 100 bp.

### Statistical analysis

Two‐tailed Student's *t*‐tests were employed to compare data from two groups, while Tukey's honestly significant difference analyses were utilized for comparing multiple groups. Both analyses were conducted using SPSS version 17.

## Conflict of interest

The authors declare no conflict of interest.

## Author contributions

L.T. conceived and designed the experiments. D.Y. and S.Z. performed most of the experiments. D.Y., D.G. and X.M. completed the bioinformatics analyses of all data. L.S., W.J., R.L. and J.Z. provided technical assistance and conducted the collection and maintenance of rice germplasm. D.Y. and L.T. performed data analysis and wrote the manuscript.

## Supporting information


**Figure S1** Graphical genotypes of *Oryza nivara* (W2014), *indica* variety 9311 and introgression line Ra139.
**Figure S2** Comparison of yield related traits between the wild type and the *mts1* mutant.
**Figure S3** The coding sequence of *MTS1* and the amino acid sequence of the MTS1.
**Figure S4** Protein structure prediction of MTS1 and mts1.
**Figure S5** Phenotype characterization of the *MTS1* knockout mutant.
**Figure S6** Phenotype characterization of the RNAi transgenic lines.
**Figure S7** Phenotype characterization of the complemented transgenic lines.
**Figure S8** The phylogenetic analysis of MTS1.
**Figure S9** Amino acid alignment among MTS1 in rice, BV1 in *Zea mays* and FRA3 in *Arabidopsis thaliana*.
**Figure S10** Measurement of PI4P concentration.
**Figure S11** Analysis of GA response in the wild type and the *mts1* mutant.
**Figure S12** Specificity detection of NGR5 antibody.
**Figure S13** Phenotype characterization of the genotypic combination of *MTS1* and *SD1*.
**Figure S14** Haplotype analysis of the *MTS1*.

**Table S1** Differentially expressed genes in the BR signal transduction pathway between the WT and the *mts1* mutant.

**Table S2** Primers used in this study.

## Data Availability

The data supporting the findings of this study are available within the article and its supplementary materials.
