# Supplementary material for: MTS1 regulates rice plant architecture by mediating phosphoinositide metabolism
Source: Plant Biotechnol J. 2025 Jul 8;23(10):4477–90. doi: 10.1111/pbi.70247 (PMC12483982; doi:10.1111/pbi.70247)
Supplement: Supplementary file 1 — Figure S1 Graphical genotypes of Oryza nivara (W2014), indica variety 9311 and introgression line Ra139. Figure S2 Comparison of yield related traits between the wild type and the mts1 mutant. Figure S3 The coding sequence of MTS1 and the amino acid sequence of the MTS1. Figure S4 Protein structure prediction of MTS1 and mts1. Figure S5 Phenotype characterization of the MTS1 knockout mutant. Figure S6 Phenotype characterization of the RNAi transgenic lines. Figure S7 Phenotype characterization of the complemented transgenic lines. Figure S8 The phylogenetic analysis of MTS1. Figure S9 Amino acid alignment among MTS1 in rice, BV1 in Zea mays and FRA3 in Arabidopsis thaliana. Figure S10 Measurement of PI4P concentration. Figure S11 Analysis of GA response in the wild type and the mts1 mutant. Figure S12 Specificity detection of NGR5 antibody. Figure S13 Phenotype characterization of the genotypic combination of MTS1 and SD1. Figure S14 Haplotype analysis of the MTS1. [file PBI-23-4477-s001.pdf]

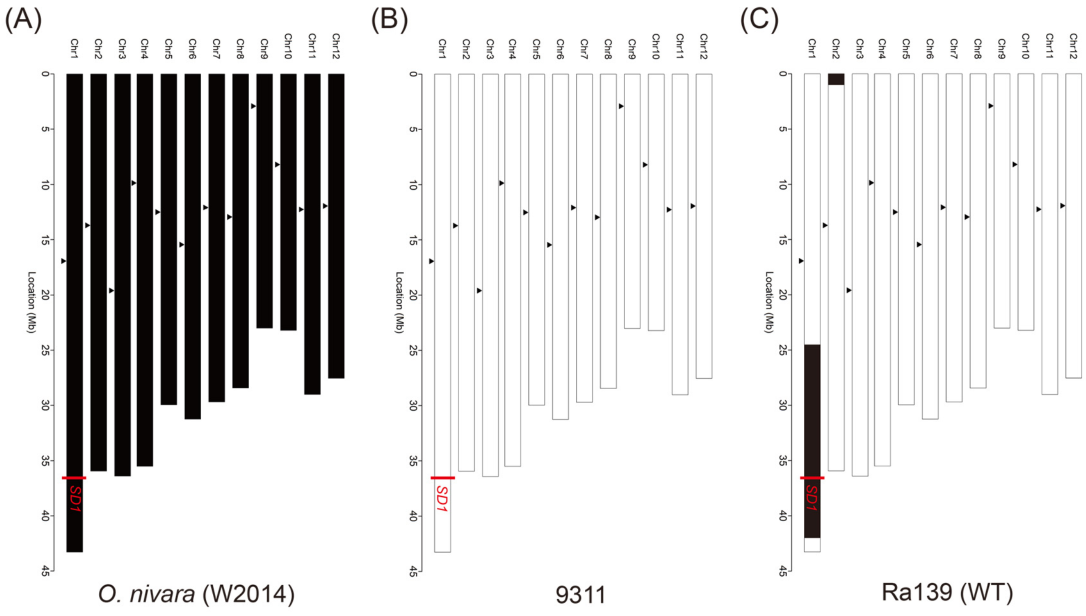


**Figure S1.** Graphical genotypes of *O. nivara* (W2014), *indica* variety 9311, and introgression line Ra139.

(A–C) The black regions indicate the regions that are homozygous for the W2014 genome. The white regions indicate the regions that are homozygous for the 9311 genome. The triangles indicate the centromere of corresponding chromosome. The red lines display where *SD1* locates in the rice genome, respectively.

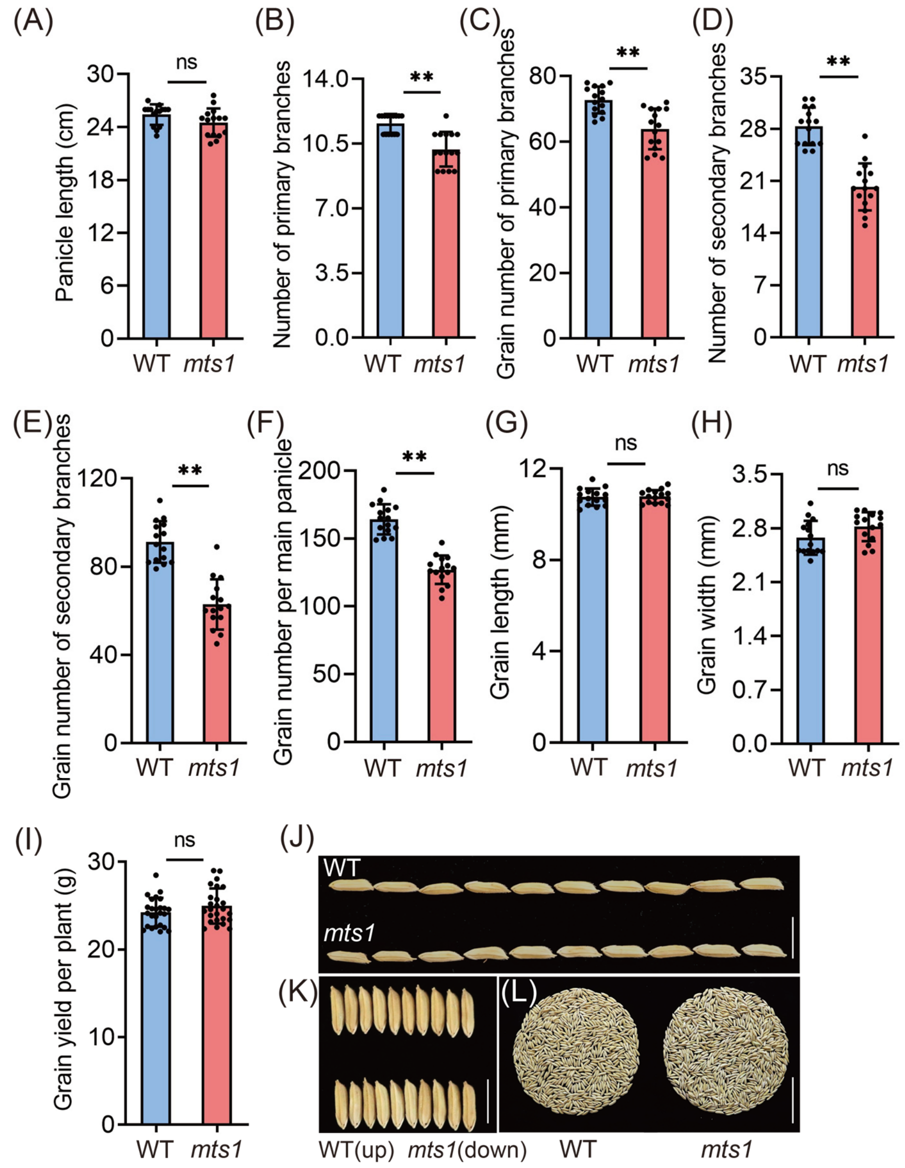


**Figure S2**. Comparison of yield related traits between the wild type and the *mts1* mutant.

(A–H) Comparison of panicle length (A), number of primary branches (B), grain number of primary branches (C), number of secondary branches (D), grain number of secondary branches (E), grain number per main panicle (F), grain length (G), and grain width (H) in the wild type (WT) and the *mts1* mutant. Data represent mean ± SD (n = 15). Two-tailed Student's *t*-test (** $P < 0.01$, ns, not significant).

(I) Comparison of grain yield per plant in in the wild type (WT) and the *mts1* mutant. Data represent mean ± SD (n = 25). Two-tailed Student's *t*-test (ns, not significant).

(J–L) Comparison of grain length (J), grain width (K), and total grains of one plant (L) in the wild type (WT) and the *mts1* mutant. Scale bas, 1 cm (J and K), 5 cm (L).

1 ATGGCGGATCCCGGCGACTCCGCCGCGTCCCCCTCCCCGTGGGACGACCTCCCCGACGAC

1 M A D P G D S A A S P S P W D D L P D D

61 TTCTTCCTCTCCGCCTCCATCTCCTCCCCTTCCCCTTCCACCTCCACTCCCCCTCCCCCT

21 F F L S A S I S S P S P S T S T P P P P

121 TCCCCCATCCCCTCCACCTCCCCGCGCCCCACCCTCCGCTCCTCCTCCCTCCCGCCCGCC

41 S P I P S T S P R P T L R S S S L P P A

181 TCCACCCCCTCCCCCTCCTCCTCTGCCTCCTCCTCCTCTGGCTCCCTCCACCACCTCCAC

61 S T P S P S S S A S S S S G S L H H L H

241 CCCACCCACTCCCTCCCGGCCTTCTCCGCCGCGGCCGCGGCGGCGGCAGCCGACGCCTGG

81 P T H S L P A F S A A A A A A A A D A W

301 CCCCCGCCACCGGGGGCGCACCACTCCGGCTCCCTCCAGGAGTTCGCCGCCCCCGCGTCC

101 P P P P G A H H S G S L Q E F A A P A S

361 TCCTCCGCGACCCGCCCGCCCCCGCGCGCCGCCGTGCGCGCCGACCGCCCGCCGCCGCTC

121 S S A T R P P P R A A V R A D R P P P L

421 GACCTGCGCCCGCGCCCGCCCAGGGAATCCCAGGCTGGCGCCGCGCTCCGCGCGATCGCC

141 D L R P R P P R E S Q A G A A L R A I A

481 GTGGACGGGGCCACCGGCTCGCACCTGTGGGCCGTCGGCGACGCCGGCGTCAGGGTGTGG

161 V D G A T G S H L W A V G D A G V R V W

541 AGCCTCGCCGACGCCTTCCGCGCGCCCGCGTCTCGGCAGAGGTGGGGCGACGAGGCTGCC

181 S L A D A F R A P A S R Q R W G D E A A

601 GCGCCGTTCCGCGAGTCGCGCAGGACGCAGCCGGCGCTGTGCCTCGTGGCTGACCCTTGC

201 A P F R E S R R T Q P A L C L V A D P C

661 CGCGGCGTTGTGTGGAGCGGCCACGCCAATGGCTGGATCATGGGGTGGAGCGCCGATCCA

221 R G V V W S G H A N G W I M G W S A D P

721 GGAGGACTAGAGGCCGGGGAGTGCATTGCCTGGGAAGCTCACCGTGGACCAGTATTCGCA

241 G G L E A G E C I A W E A H R G P V F A

781 TTGACCACCTCTCTATATGGTGATTTGTGGTCTGGCTCTGAAGGGGGAGTAATCAAAGTT

261 L T T S L Y G D L W S G S E G G V I K V

841 TGGTATGAAGAAGGAATTGAGAAGTCCCTTTCTTTACAAAGAGAAGAAAAGCGTAAGACC

281 W Y E E G I E K S L S L Q R E E K R K T

901 TCCTTTTTAGTTGAAAGATCATTCATTGACCTTAGAGCCATGGTCAGTGATGGAGGGGCC

301 S F L V E R S F I D L R A M V S D G G A

961 TGTCCCTTGCCTGCCGTAGATGTAAAACTTCTGTTGTCTGACAATTCTAGATCAAAAGTA

321 C P L P A V D V K L L L S D N S R S K V

1021 TGGAGTGCTGGTTACCTCTCACTTGCACTCTGGGATTCTTGCACCAAGGAGCTTCTGAAA

341 W S A G Y L S L A L W D S C T K E L L K

1081 GTGATTAGCGTGGATGGCCAAGTCGATACTCGTTTTGATATCTTATCTTCTCAGGATCCA

361 V I S V D G Q V D T R F D I L S S Q D P

1141 TTTGGCTATGAAACAAAGCAAAACCTTTTCTCTGCTCCAAGGAAAGATAAAGCTCGCAGT

381 F G Y E T K Q N L F S A P R K D K A R S

C in *mts1*

1201 CCTGTTGGATTTTTCCAGAGATCACGAAATGCTTTAATGGGAGCGGCTGATGCAGTCCGG

401 P V G F F Q R S R N A L M G A A D A V R

S in *mts1*

1261 AGAGTTGCTGCTAAAGCAGGATTTGGAGATGATTCTCAAAGAATAGAAGCATTAGCAATG
421 R V A A K A G **F G D D S Q R I E A L A M**
1321 TCAATTGATGGGATGATCTGGACAGGGTCTGCAAATGGATGTCTTGCTCGATGGGATGGC
441 **S I D G M I W T G S A N G C L A R W D G**
1381 AACGGTAACCGTTTGCAAGAGTTTCAGCATCATTTGTGTTCTGTTCAAAGCATTTTCAGC
461 **N G N R L Q E F Q H H L C S V Q S I F S**
1441 TTTGGGACAAGAATATGGGCCGGTTACATGGATGGTAGTATTCAGCTGTTGGACTTGGAA
481 **F G T R I W A G Y M D G S I Q L L D L E**
1501 GGTAACTTACTAGGAGGCTGGATCGCACATAGCAGTCCAGTTCTGAGTATGGCTGTTGGA
501 **G N L L G G W I A H S S P V L S M A V G**
1561 GGTTCATACATCTTTACAATGGCTGGTCATGGCGGAGTCCGTGGATGGAATTTGTCATCT
521 **G S Y I F T M A G H G G V R G W N L S S**
1621 CCAGGGCCTATTGACAACATTATGCGTTCTACTTTGATTGAGGCTGAGCCATTATACAAA
541 **P G P I** D N I M R S T L I E A E P L Y K
1681 CAATTTGAATACATGAAAGTGTTGGTGGGTTCTTGGAATGTCGGGCAAGAAAAGGCATCT
561 Q F E Y M **K V L V G S W N V G Q E K A S**
1741 TATGAGTCACTAAGAGCTTGGCTAAAGTTACCGACACCAGAGGTTGGGTTAGTGGTAGTT
581 **Y E S L R A W L K L P T P E V G L V V V**
1801 GGATTGCAGGAGGTGGACATGGGTGCTGGTTTTCTTGCAATGTCTGCAGCTAAAGAAACA
601 **G L Q E V D M G A G F L A M S A A K E T**
1861 GTTGGGCTAGAGGGAAGCCCAAACGGAGATTGGTGGTTGGATGCAATTGGGCAGCAGTTA
621 **V G L E G S P N G D W W L D A I G Q Q L**
1921 AAGGGTTACTCTTTTGAGCGTGTTGGCTCGAGGCAGATGGCTGGATTGCTTATCTGTGTA
641 **K G Y S F E R V G S R Q M A G L L I C V**
1981 TGGGTCAGAACACATCTTAAGCAGTTCATTGGTGATATTGATAATGCTGCGGTAGCATGT
661 **W V R T H L K Q F I G D I D N A A V A C**
2041 GGATTAGGGCGAGCAATCGGCAACAAGGGAGCAGTGGGATTGAGGATGAGAATACATGAT
681 **G L G R A I G N K G A V G L R M R I H D**
2101 AGGAGTATTTGCTTTGTAAATTGCCATTTTGCTGCTCATATGGAAGCTGTGAGTCGACGG
701 **R S I C F V N C H F A A H M E A V S R R**
2161 AATGAAGATTTTGACCATGTCTTTAGAACAATGACCTTTGCCACCCCTTCGAGTGGAATA
721 **N E D F D H V F R T M T F A T P S S G I**
2221 ATGACAACATCAGTTTCTAGTTCTACTGGCCAGCTTCTTCGAGGAGCAAATGGATCAAGA
741 **M T T S V S S S T G Q L L R G A N G S R**
2281 ATGCCTGAGTTGTCAGACACGGACATGATTGTCTTTCTTGGTGACTTCAATTACCGCCTT
761 **M P E L S D T D M I V F L G D F N Y R L**
2341 TATGATATTTCCTATGATGATGCAATGGGCTTAGTTTCCCGGAGATGCTTTGACTGGCTA
781 **Y D I S Y D D A M G L V S R R C F D W L**
2401 AAAAATAATGACCAACTGCGAGCAGAAATGAGATCTGGGAGAGTCTTCCAGGGACTACGT
801 **K N N D Q L R A E M R S G R V F Q G L R**
2461 GAAGGGGATTTCAAGTTTCCCCCTACATACAAATTTGAGAAACATACAGCAGGCTTATCA
821 **E G D F K F P P T Y K F E K H T A G L S**
2521 GGGTATGATAGCAGTGAGAAGAGGCGCATTCCTGCCTGGTGTGACAGAATCCTATATCGT
841 **G Y D S S E K R R I P A W C D R I L Y R**

2581 GATAGCCGAGTTAGTTCAGGGAATGAGTGTTCCTTGGATTGTCCTGTGGTTTCTTCAATA

861 **D S R V S S G N E C S L D C P V V S S I**

2641 TCACTGTATGACTCTTGCATGGAAGCAACAGATAGTGATCACAAACCTATAAAATCTGTG

881 **S L Y D S C M E A T D S D H K P I K S V**

2701 TTCAATTTGGATATTGCTTATGTTGACAAACAGACAATGAGGCAGAAATATGTGGAGCTA

901 **F N L** D I A Y V D K Q T M R Q K Y V E L

2761 ATGAGCTCAAATAATAAAGTGGTGCATTTGCTTCAGGAACTTGAAGCATTCCCTGGAGTA

921 M S S N N K V V H L L Q E L E A F P G V

2821 AATATAAATAATTCTAACATCATCTTGCAAGATCGGAATCCATCTGTTGTGAAATTGCAA

941 N I N N S N I I L Q D R N P S V V K L Q

2881 AACAGAACAGAAGTCATCGCTTGTTTTGAGATCATTGGACAAGCACCAAATTTGTCCAGC

961 N R T E V I A C F E I I G Q A P N L S S

2941 ACACATTTCTCTGCTTTTCCTGCATGGCTAAAGGTCTCTCCAGCAGTCGGCATAATATCT

981 T H F S A F P A W L K V S P A V G I I S

3001 CCGGGACAGACGGTAGAGGTCACTTTGCAGCACAGAGACCTGCATAGCCAACAAAACTAT

1001 P G Q T V E V T L Q H R D L H S Q Q N Y

3061 AATGGAACTTCATTGGATATTTTGCCTGGTGGAGCTACCCAACAAAAGGCAGCAACTGTT

1021 N G T S L D I L P G G A T Q Q K A A T V

3121 TTTGCGAAAATAACTGGAGTATATTCAACAGTTGCAAAATATTACGAAATACATGTACAA

1041 F A K I T G V Y S T V A K Y Y E I H V Q

3181 CACCAGAACTGCAGGAGCACATTGCCATCGAGAGGTTATAACTTAGGTGACCGGTTTTTT

1061 H Q N C R S T L P S R G Y N L G D R F F

3241 TAA

1081 *

**Figure S3.** The coding sequence of *MTS1* and the amino acid sequence of the MTS1 protein.

The red letters indicate the nucleotide and amino acid substitutions in the *mts1* mutant.

The WD40 and EEP domain are marked in bold and underlined.

(A)

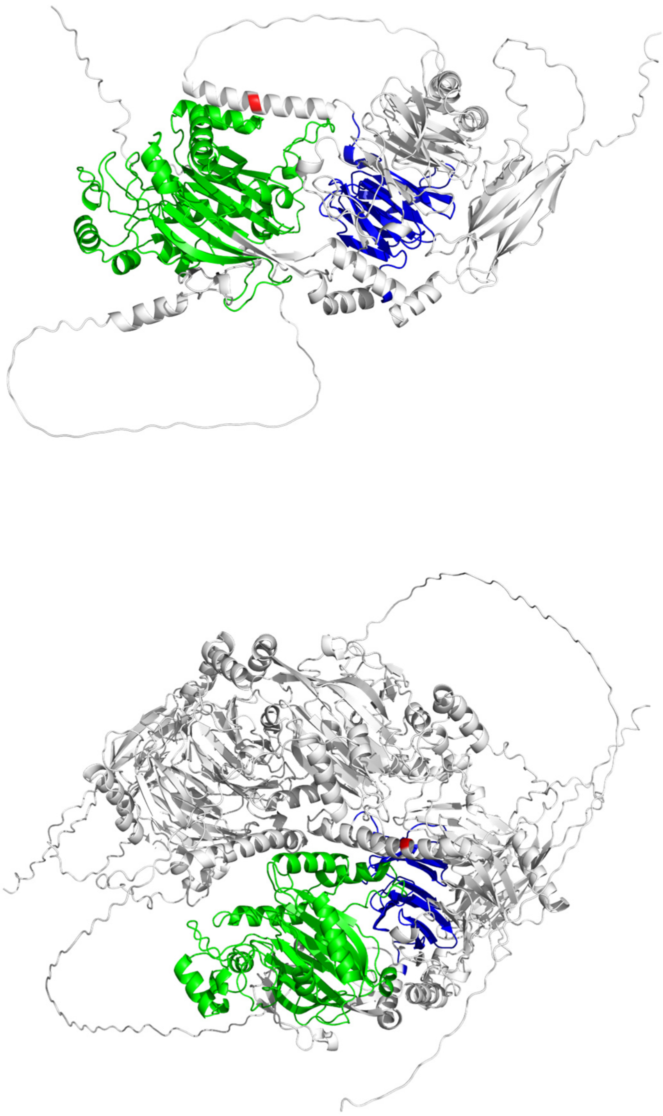

(B)

**Figure S4.** Protein structure prediction of MTS1 and mts1.
(A–B) The 3D conformation of protein MTS1 (A) and protein mts1 (B) was predicted using AlphaFold. The WD40 domain is depicted as blue, EEP superfamily domain is depicted as green and the mutation site is depicted as red.

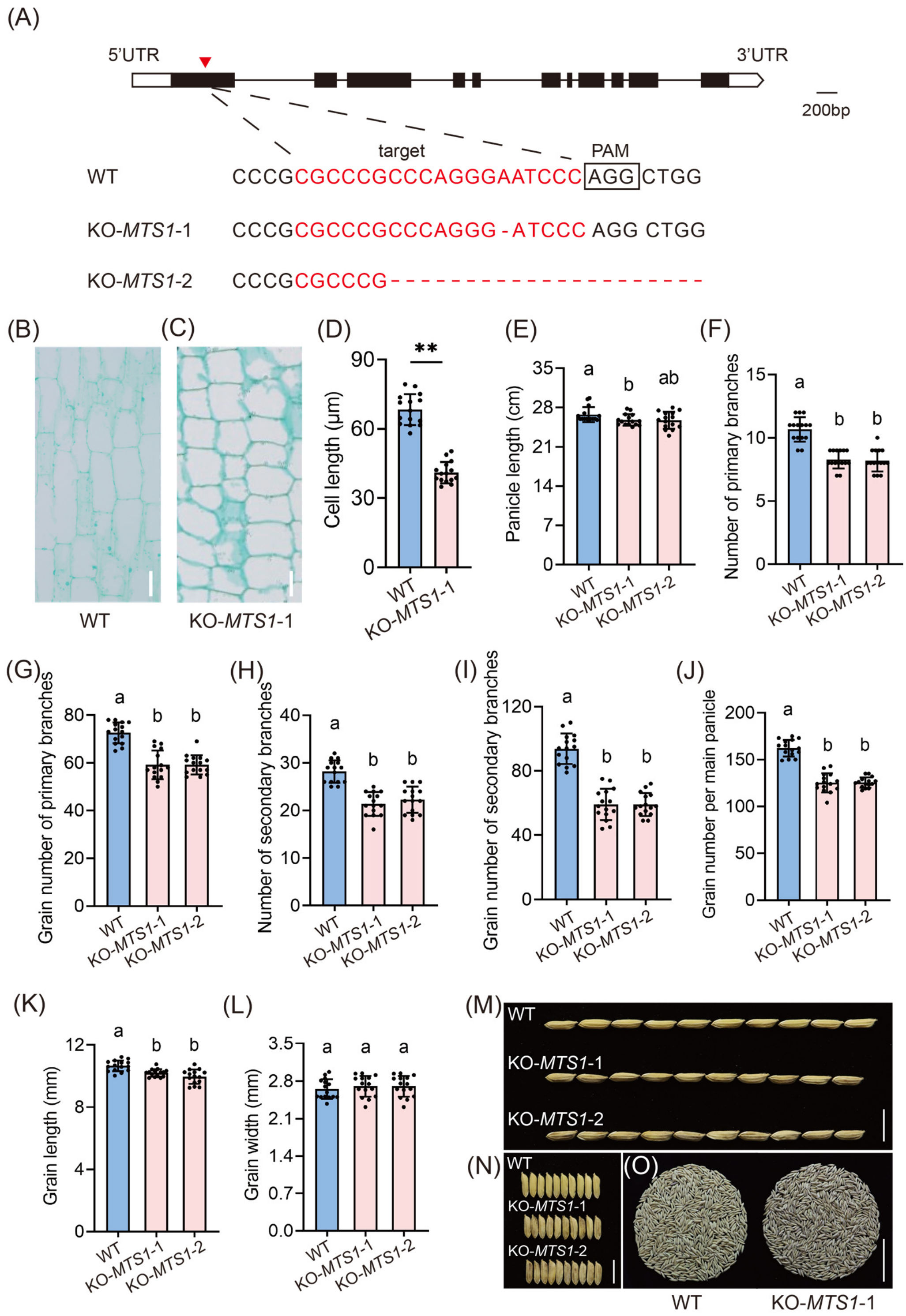


**Figure S5.** Phenotype characterization of the *MTS1* knockout mutant.

(A) Schematic illustration and sequence alignment of the sgRNA target site of *MTS1* in the knockout lines. The sgRNA target sequences are highlighted with red font and the black box indicates protospacer-adjacent motif (PAM) sequences. Deleted nucleotides are depicted as dashes.

(B and C) Longitudinal sections of the fourth internode of the wild type (B) and the

knockout mutant KO-*MTS1*-1 (C). Scale bars, 20 μm.

(D) Comparison of the length of internode cell in the wild type (WT) and the knockout mutant (KO-*MTS1*-1). Data represent mean ± SD (n = 15). Two-tailed Student's *t*-test (** $P < 0.01$).

(E–L) Comparison of panicle length (E), number of primary branches (F), grain number of primary branches(G), number of secondary branches (H), grain number of secondary branches (I), grain number per main panicle (J), grain length (K), and grain width (L) in the wild type (WT) and knockout lines (KO-*MTS1*-1 and KO-*MTS1*-2). Data represent mean ± SD (n = 15). Different lowercase letters denote significant differences (ANOVA, $P < 0.05$).

(M–O) Comparison of grain length (M), grain width (N), and total grains of one plant (O) in the wild type (WT) and knockout lines (KO-*MTS1*-1 and KO-*MTS1*-2). Scale bas, 1 cm (M and N), 5 cm (O).

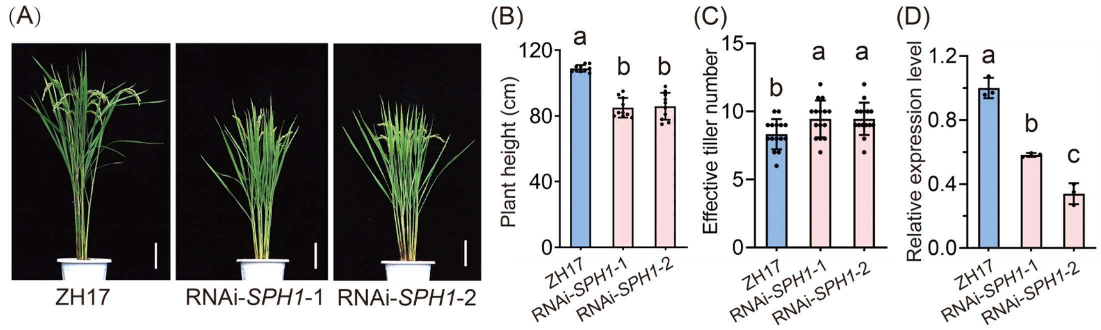


**Figure S6.** Phenotype characterization of the RNAi transgenic lines.

(A) Plant morphology of the control (ZH17) and RNAi transgenic lines (RNAi-*MTS1*-1 and RNAi-*MTS1*-2). Scale Bars, 15 cm.

(B and C) Comparison of plant height (B) and effective tiller number (C) in the control (ZH17), RNAi-*MTS1*-1 and RNAi-*MTS1*-2. Data represent mean ± SD (n = 10). Different lowercase letters denote significant differences (ANOVA, $P < 0.05$).

(D) Comparison of relative expression level in the control (ZH17) and RNAi transgenic lines (RNAi-*MTS1*-1 and RNAi-*MTS1*-2). Data represent mean ± SD (n = 3). Different lowercase letters denote significant differences (ANOVA, $P < 0.05$).

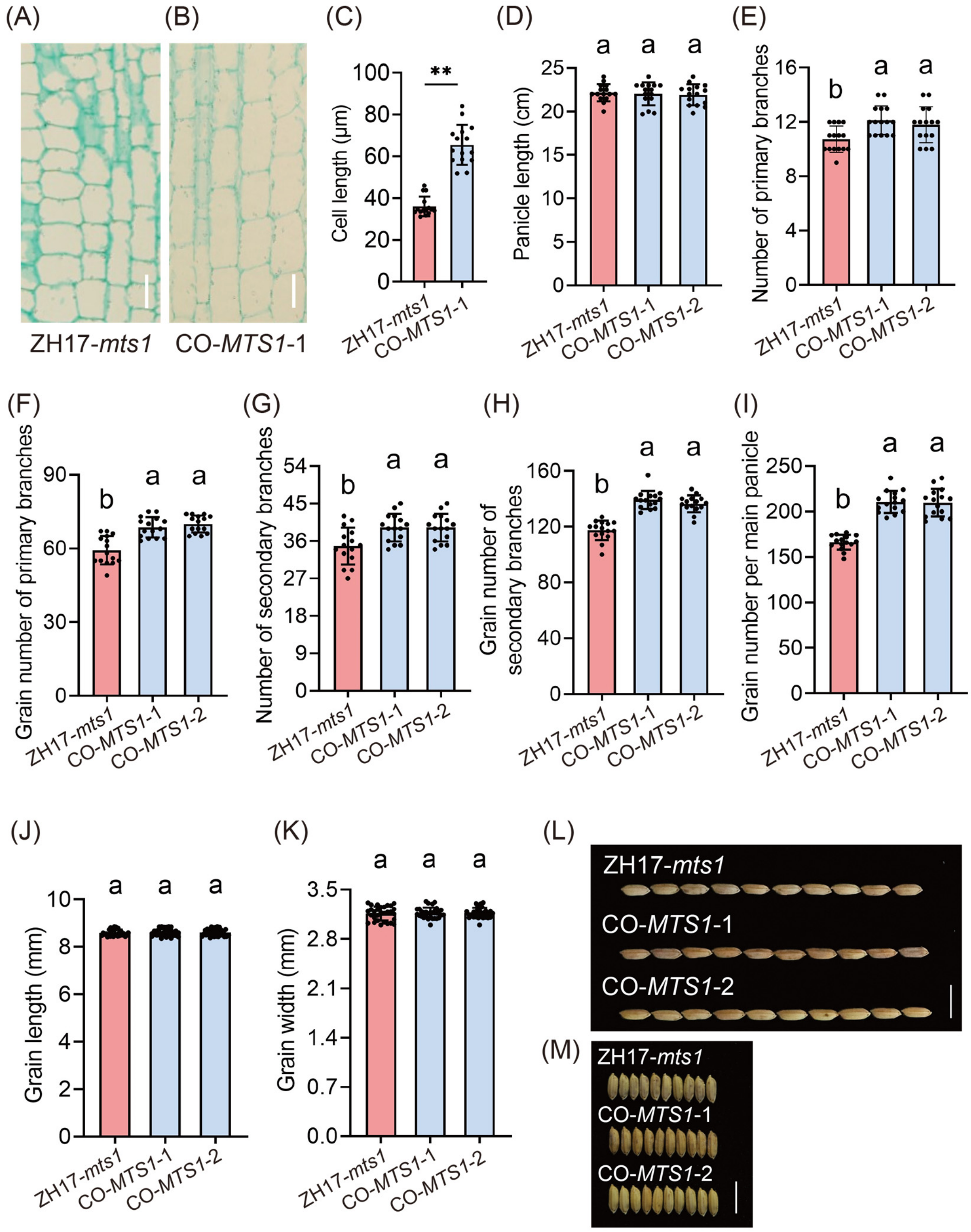


**Figure S7.** Phenotype characterization of the complemented transgenic lines.

(A and B) Longitudinal sections of the fourth internode of the ZH17-*mts1* (A) and the complemented transgenic lines (CO-*MTS1*-1) (B). Scale Bars, 20 μm.

(C) Comparison of the length of internode cell in the ZH17-*mts1* and the complemented transgenic lines (CO-*MTS1*-1). Data represent mean ± SD (n = 15). Two-tailed Student's *t*-test (** $P < 0.01$).

(D–K) Comparison of panicle length (D), number of primary branches (E), grain number of primary branches (F), number of secondary branches (G), grain number of secondary branches (H), grain number per main panicle (I), grain length (J), and grain width (K) in the ZH17-*mts1* and the complemented transgenic lines (CO-*MTS1*-1 and CO-*MTS1*-2). Data

represent mean ± SD (n = 15). Different lowercase letters denote significant differences (ANOVA, $P < 0.05$).

(L and M) Comparison of grain length (L) and grain width (M) in the ZH17-*mts1* and the complemented transgenic lines (CO-*MTS1*-1 and CO-*MTS1*-2). Scale Bars, 1 cm.

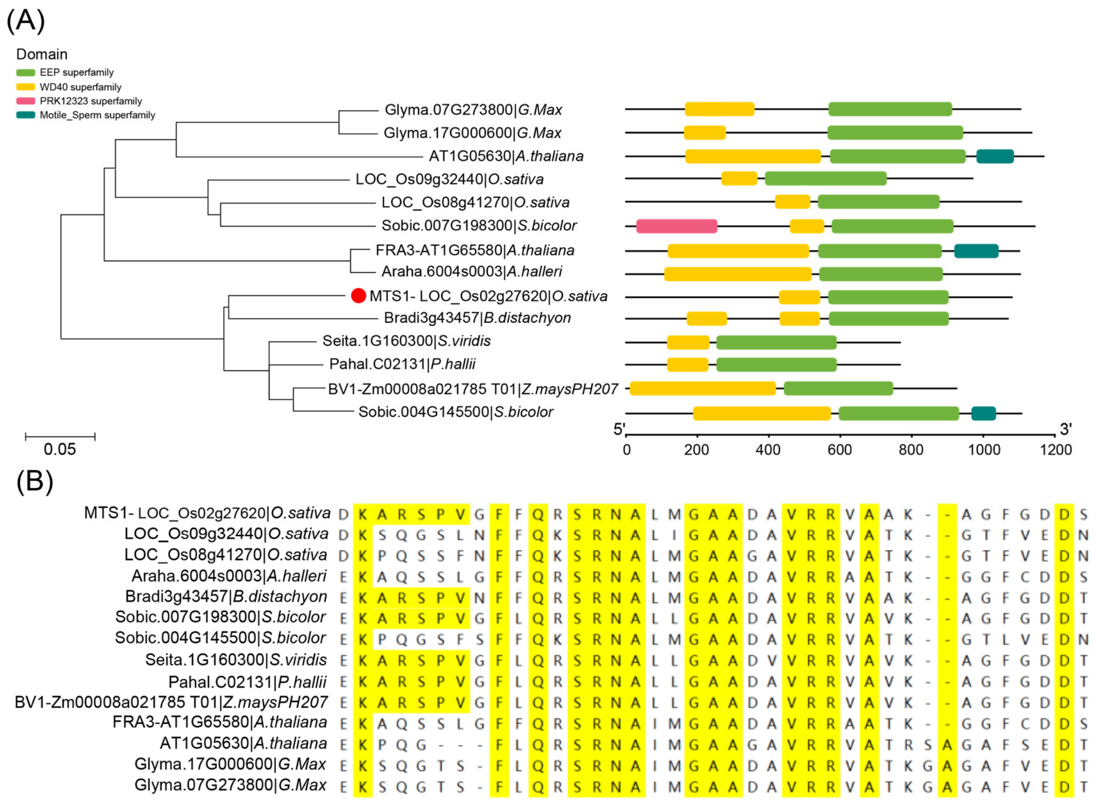


**Figure S8.** The phylogenetic analysis of MTS1.

(A) Phylogenetic tree of MTS1 protein in plant species.

(B) Partial amino acid sequence alignment of MTS1 and its homologs. The red box indicates the location of amino acid substitution site from WT (L) to *mts1* (S).

```
MTS1 : MADPGDSAASPSPWDDLPDDFFLSASISSPSPSTSTPPPPSPIPSTSPRPTLRSSSLPPA :   60
BV1  : MANPG---------------------------------------GTNP------------ :    9
FRA3 : MEDRQN---------DQNDDVFSFFSPSFSAATPSTLFNRSAYSSSSS-------SGDDE :   44

MTS1 : STPSPSSSASSSSGSLHHLHPTHSLPAFSAAAAAAAADAWPPPPGAHHSGSLQEFAAPAS :  120
BV1  : -FASPADAASS---------------------------------PESQAGAALRVLACCCD :   36
FRA3 : SQPSVDDSNKRIDYMIQFLDRRLSEDGNHDG-----------IGDGNGSDSLPEFVGKCG :   93

MTS1 : SS-ATRPPPRAAVRADRPPPLDLRPRPPRESQAGAALRAIAVDGATGSHLWAVG-DAGVR :  178
BV1  : AS-RSEP--------------------------GAAAR-----------LWAAG-EAGVR :   57
FRA3 : ESGIFKVPIRSAVHPNRPPSLDVRPHPLRETQIGRFLRTMTS---TERQLWTGGEDGALR :  150

MTS1 : VWSLADAFRAPASRQRWGDEAAAPFRESR----RTQPALCLVADPCRGVVWSGHANGWIM :  234
BV1  : AWDLADAFRSSASQQRWGDESSAPFRESR----RTPPALCLAADPARSVVWSGHADGRIM :  113
FRA3 : VWEFSELY---GSGRGLEVEDTAPYKESLGNEFGSAAVVCMIGDEGSRVVWSGHRDGRIR :  207

MTS1 : GWSADPGGLEAGECIAWEAHRGPVFALTTSLYGDLWSGSEGGVIKVW-YEEGIEKSLSLQ :  293
BV1  : GWTADPG-PEAGECLSWEAHRGPVFALAVSSYGDLWSGSEGGAIKVW-YAELIQKSLVSQ :  171
FRA3 : CWRLRGD-HGIEEALSWQAHRGPVLSIAISAYGDIWSGSEGGALKVWPWDGALGKSLSLK :  266

MTS1 : REEKRKTSFLVERSFIDLRAMVSDGGACPLPAVDVKLLLSDNSRSKVWSAGYLSLALWDS :  353
BV1  : TEEKRK-ALLVERSSVDLRDMVSDGGACPLPAVDVKLLLSDNSRSKVWSAGYLSFALWDS :  230
FRA3 : MEERHMAALAVERSYIDPRNMVSANGFANTLTSDVTFLVSDHTRARVWSASPLTFAIWDA :  326

MTS1 : CTKELLKVISVDGQVDTRFDILSSQDPFGYETKQNLFSAPRKDKARSPVGFFQRSRNALM :  413
BV1  : RTKGLLKVVNIDGQVDTRFDILSAQDSYSYETKQTLFS-PRKEKARSPVGFLQRSRNALL :  289
FRA3 : RTRDLIKVFNIDGQLENRPENSVYPDFGSEEEGKMKVTASKKEKAQSSLGFFQRSRNAIM :  386

MTS1 : GAADAVRRVAAKAGFGDDSQRIEALAMSIDGMIWTGSANGCLARWDGNGNRLQEFQHHLC :  473
BV1  : GAADAVRRVAVKAGFGDDTRRIEAFTMSTDGMIWTGSANGSLAQWDGSGNRLQEFLHHSS :  349
FRA3 : GAADAVRRAATKGGFCDDSRKTEAIVISVDGMIWTGSSNGILMRWDGNGNCLQEFAYESS :  446

MTS1 : SVQSIFSFGTRIWAGYMDGSIQLLDLEGNLLGGWIAHSSPVLSMAVGGSYIFTMAGHGGV :  533
BV1  : SVQCIYNFGTRIWVGYMDGNIQLLDLEGNLLGGWIAHSSPILSMAVGGSYIFTLAGHGEI :  409
FRA3 : GILCMFTFCSRLWVGYSNGTVQVWDLEGKLLGGWVAHSGPVIKMAIGAGYLFTLANHGGI :  506

MTS1 : RGWNLSSPGPIDNIMRSTLIEAEPLYKQFEYMKVLVGSWNVGQEKASYESLRAWLKLPTP :  593
BV1  : RGWNLASPGPLDNILRSELMEKDSSYKSFEYMKVLVGSWNVGQEKASYESLRAWLKLPSP :  469
FRA3 : RGWNVTSPGPLDNVLRAELAGKEFLYSRIENLKILAGTWNVGEGRASTDSLVSWLGCAAT :  566
```

```
MTS1 : EVGLVVVGLQEVDMGAGFLAMSAAKETVGLEGSPNGDWWLDAIGQQLK-GYSFERVGSRQ :  652
BV1  : EVGLVVLGLQEVDMGAGFLAMSAAKETVGLEGSTNGEWWLDVIGQILK-VHSFVRVGSRQ :  528
FRA3 : GVEIVVVGLQEVEMGAGVLAMSAAKETVGLEGSPLGQWWLDMIGKTLDEGSSFVRVGSRQ :  626

MTS1 : MAGLLICVWVRTHLKQFIGDIDNAAVACGLGRAIGNKGAVGLRMRIHDRSICFVNCHFAA :  712
BV1  : MAGLLIAVWVRINLKQFIGDIENAAVPCGLGRAIGNKGAVGLRMRIHGRNICFVNCHFSA :  588
FRA3 : LAGLLICVWVRHDLKPHVGDVDAAAVPCGFGRAIGNKGAVGVRLRMYDRVLCFVNCHFAA :  686

MTS1 : HMEAVSRRNEDFDHVFRTMTFATPSSGIMTTSVSSSTGQLLRGANGSRM--------PEL :  764
BV1  : HMEAVSRRNEDFDH-------------------------------GSRL--------PEL :  609
FRA3 : HLEAVNRRNADFDHVYRTMTFSRQSSSLNAGVAGASFG-VTMPRGGNALGVNTIEARPEL :  745

MTS1 : SDTDMIVFLGDFNYRLYDISYDDAMGLVSRRCFDWLKNNDQLRAEMRSGRVFQGLREGDF :  824
BV1  : SDTDLIVFLGDFNYRLYNISFDEAMGLVSRRCFDWLRDNDQLRAEMKSGRVFQGLREGEF :  669
FRA3 : SEADMVIFLGDFNYRLDDITYDETRDFISQRCFDWLREKDQLHTEMEAGNVFQGMREAII :  805

MTS1 : KFPPTYKFEKHTAGLSGYDSSEKRRIPAWCDRILYRDSRVSSGNECSLDCPVVSSISLYD :  884
BV1  : KFPPTYKFEKHIAGLSGYDNSEKRRIPAWCDRVLYRDSRTSSQIECSLECPVVCSISLYD :  729
FRA3 : RFPPTYKFERHQAGLAGYDSGEKKRIPAWCDRILYRDNKKHLGAECSLDCPVVSSISQYD :  865

MTS1 : SCMEATDSDHKPIKSVFNLDIAYVDKQTMRQKYVELMSSNNKVVHLLQELEAFPGVNINN :  944
BV1  : SCMEATDSDHKPVKCVFNLDIAHVDKQTMRQKYGEIMSSNKEVLDSLQGLEALPEVDIST :  789
FRA3 : ACMEVTDSDHKPVRCVFSVKIARVDESVRRQEYGNIINSNKKIKVLLGELSKVPETIVST :  925

MTS1 : SNIILQDRNPSVVKLQNR-TEVIACFEIIG----------QAPNLSSTHFSAFPAWLKVS :  993
BV1  : NDIMLQDQNPFVVKLHNRSTKELACFEIIG----------QTPMSSGTAFSGFPSWLKVS :  839
FRA3 : NNIILQNQDSTILRITNKSEKNIAFFKIICEGQSKIEEDGQAHDHRARGSFGFPQWLEVS :  985

MTS1 : PAVGIISPGQTVEVTLQHRDLHSQQNYNGTSLDILPGGATQQKAATVFAKITGVYSTVAK : 1053
BV1  : PAVGIISPRQSVEVTLQHGQIRSQDYLTGTSGDSS--GAAQEKVATLLVTVTRVDSTAGR :  897
FRA3 : PGTGTIKPNQIAEVSVHLEDFPTVEEFVDGVAQNSWCEDTRDKEVILVLVVHGRFSTETR : 1045

MTS1 : YYEIHVQHQNCR-----------------STLPSRGYNLGDRFF------------ : 1080
BV1  : RHKIQVQHRCRR-----------------ETYSSRGYNLADRFFA----------- :  925
FRA3 : KHRIRVRHCPRGGPAKNHFNDGTKTSGQINALHRSDYHQLSNTLDVVEQLKNLHSP : 1101
```

**Figure S9.** Amino acid alignment among MTS1 in rice, BV1 in *Zea mays* and FRA3 in *Arabidopsis thaliana*.

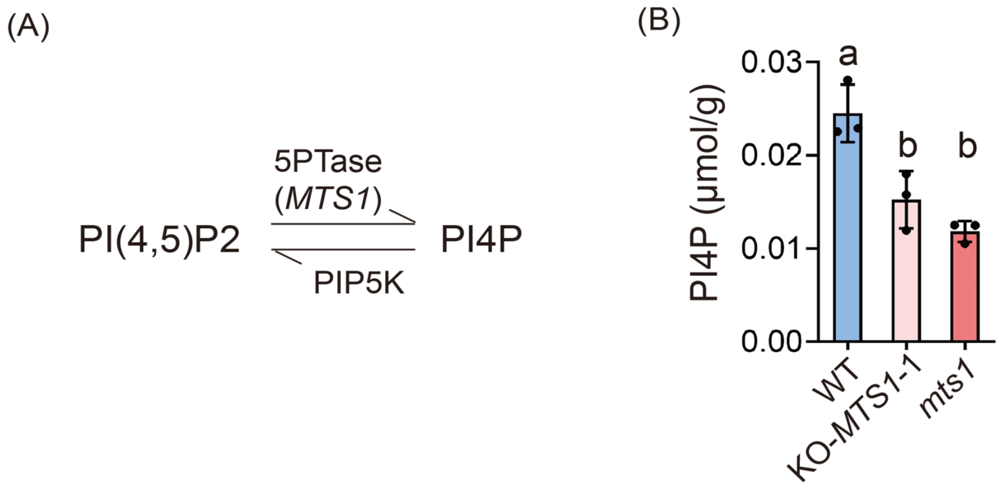


**Figure S10.** Measurement of PI4P concentration.

(A) 5PTase hydrolyzes PI(4,5)P2 to PI4P.

(B) Comparison of PI4P concentration among the wild type (WT), knockout mutant (KO-*MTS1*-1), and the *mts1* mutant. Data represent means ± SD (n = 3). Different lowercase letters denote significant differences (ANOVA, $P < 0.05$,).

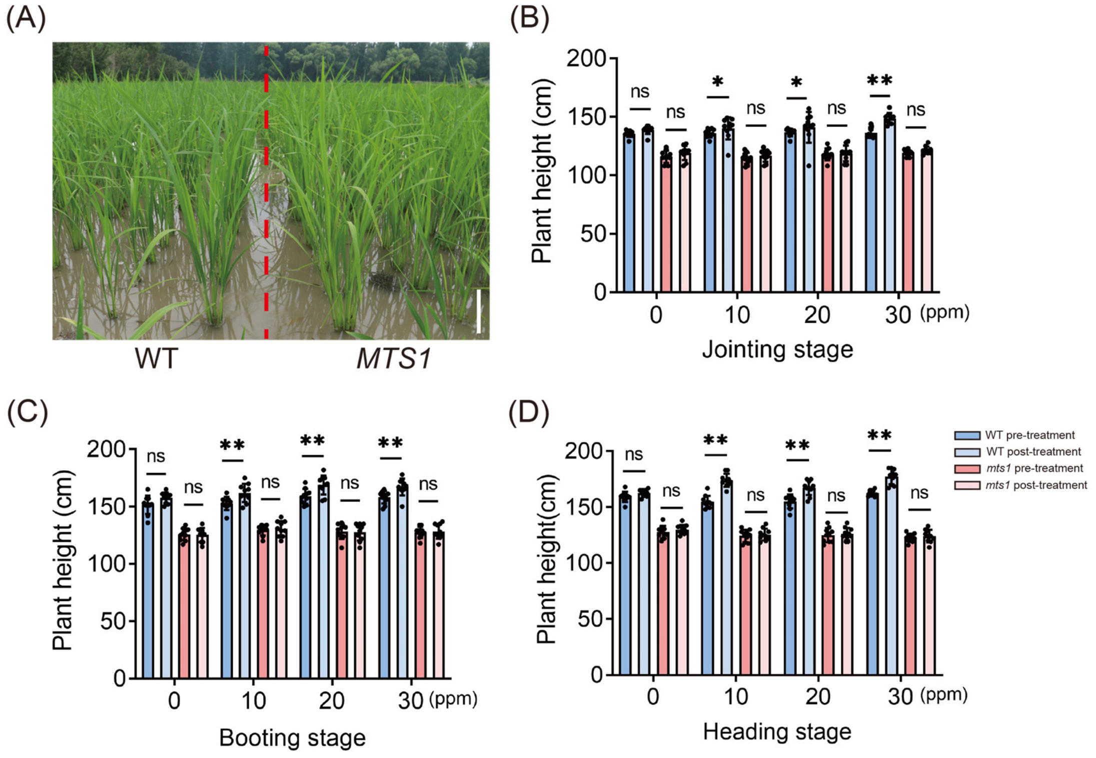


**Figure S11.** Analysis of GA response in the wild type and the *mts1* mutant.

(A) Plant morphology of the wild-type and the *mts1* mutant at the tillering stage in the field. Scale bar, 10 cm.

(B–D) Comparison of plant height in the wild type (WT) and the *mts1* mutant before and after the treatments using different concentrations of $GA_3$ at the jointing stage (B), the booting stage (C), and the heading stage (D). Data represent mean ± SD (n = 10). Two-tailed Student's *t*-test (** $P < 0.01$; ns, not significant).

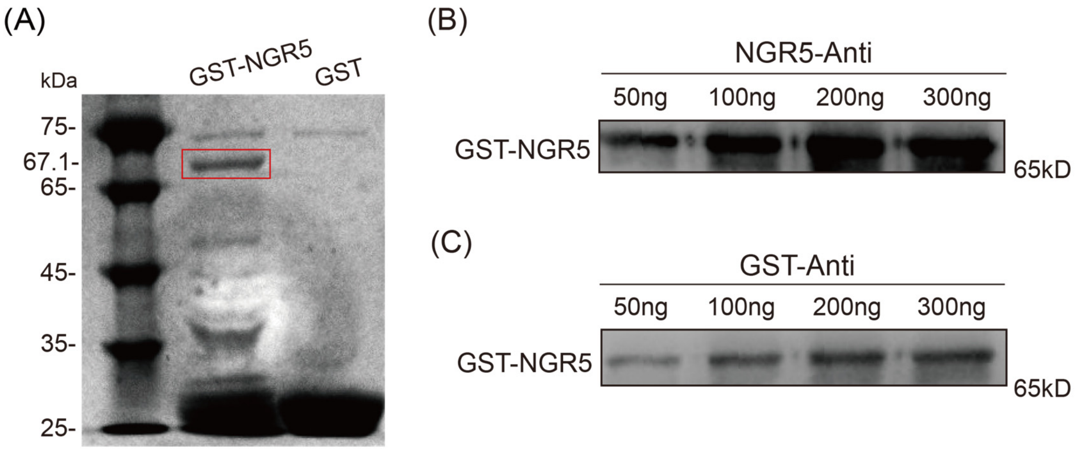


**Figure S12.** Specificity detection of NGR5 antibody.

(A) The gel stained with Coomassie gray and the molecular weight of GST-NGR5 is 67.1 kDa.

(B) The binding of NGR5 autoantibodies to different amounts of GST-NGR5.

(C) The binding of GST antibody to different amounts of GST-NGR5.

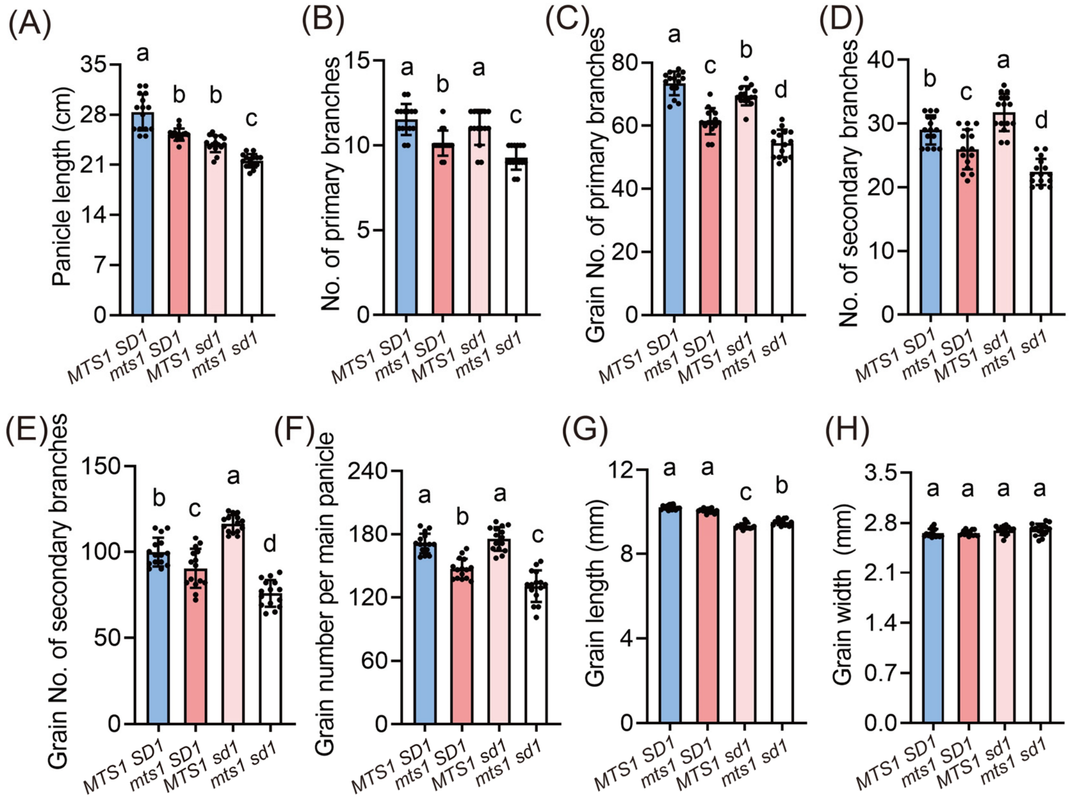


**Figure S13.** Phenotype characterization of the genotypic combination of *MTS1* and *SD1*. (A–H) Statistical analysis of panicle length (A), number of primary branches (B), grain number of primary branches (C), number of secondary branches (D), grain number of secondary branches (E), grain number per main panicle (F), grain length (G), and grain width (H) of *MTS1 SD1*, *mts1 SD1*, *MTS1 sd1* and *mts1 sd1*. Data represent mean ± SD (n = 15). Different lowercase letters denote significant differences (ANOVA, $P < 0.05$).

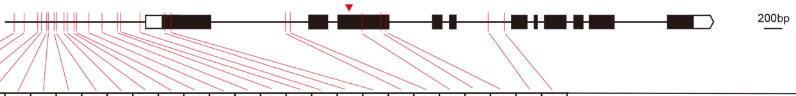


| Haplotype \ Position | 17106389 | 17106477 | 17106626 | 17106654 | 17106694 | 17106702 | 17106752 | 17106770 | 17106883 | 17106904 | 17107000 | 17107024 | 17107203 | 17107391 | 17107753 | 17107794 | 17107957 | 17108451 | 17108586 | 17109851 | 17109893 | 17110834 | 17111206 | 17111236 | 17112592 | 17112763 | No. of accessions | | | |
|---|---|---|---|---|---|---|---|---|---|---|---|---|---|---|---|---|---|---|---|---|---|---|---|---|---|---|---|---|---|---|
| | | | | | | | | | | | | | | | | | | | | | | | | | | | *O. nivara* | *O. rufipogon* | *indica* | *japonica* |
| Hap1 | G | G | T | A | A | T | T | C | T | T | T | C | C | G | A | T | A | T | C | G | G | G | T | G | G | C | 35 | 0 | 314 | 8 |
| Hap2 | G | G | T | A | G | T | T | C | T | C | T | C | C | A | A | C | T | T | C | G | C | G | T | A | G | C | 29 | 34 | 238 | 0 |
| Hap3 | A | C | C | T | A | C | C | G | C | C | C | T | T | G | G | C | T | A | T | A | C | A | C | A | C | A | 0 | 2 | 20 | 271 |
| Hap4 | G | C | C | T | G | C | C | C | C | C | C | T | T | G | G | C | T | T | T | G | C | A | C | A | C | A | 0 | 25 | 0 | 0 |
| Hap5 | G | G | T | A | G | T | T | C | T | C | T | C | C | G | A | C | T | T | C | G | C | G | T | A | G | C | 37 | 10 | 1 | 0 |
| Hap6 | G | G | C | T | G | C | C | C | C | C | C | C | C | G | G | C | T | T | T | G | C | G | C | A | C | A | 0 | 34 | 0 | 0 |
| Hap7 | G | G | C | T | G | C | C | C | C | C | C | C | C | G | G | C | T | T | T | G | C | G | C | A | C | C | 51 | 8 | 2 | 22 |
| Hap8 | G | C | C | T | G | C | C | C | C | C | C | C | C | G | G | C | T | T | T | G | C | G | C | A | C | A | 0 | 17 | 0 | 0 |
| Hap9 | G | G | T | A | A | T | T | C | T | C | T | C | C | G | A | T | A | T | C | G | G | G | T | G | G | C | 4 | 1 | 0 | 0 |
| Hap10 | G | G | C | T | G | C | T | C | C | C | C | C | C | G | G | C | T | T | T | G | C | G | C | A | C | C | 1 | 3 | 0 | 0 |

**Figure S14.** Haplotype analysis of the *MTS1*.

Haplotype analysis of the *MTS1* in rice, based on 26 variations, the 1167 accessions (134 *O. rufipogon*, 157 *O. nivara*, 301 *japonica*, and 575 *indica* accessions) could be classified into ten haplotypes.
